# Anomaly Detection and Data Repair for Smart Meter Data in Smart Cities: A Comprehensive Review and Future Perspectives

**DOI:** 10.3390/s26165122

**Published:** 2026-08-13

**Authors:** Bensong Zhang, Guoying Lin, Kaihong Zheng, Jinyang Du

**Affiliations:** Southern Grid Digital Grid Group Co., Ltd., Guangzhou 510700, China; gaochuangxin@stu.ouc.edu.cn (G.L.); zhengkh@csg.cn (K.Z.); dujy@im.csg (J.D.)

**Keywords:** smart meter, data quality, anomaly detection, data repair, physical constraints, distribution network, line loss management

## Abstract

Smart meters are the core terminals for distribution network data acquisition in smart cities, yet their collected data commonly suffer from quality issues caused by harsh operating environments, communication failures, hardware degradation, and human factors. This paper presents a systematic review of anomaly detection and data repair methods for smart meter data based on a critical analysis of many publications. First, we characterize five typical anomalies—sudden jumps, reading stagnation, reverse readings, pulse spikes, and gradual drifts—from physical root causes to data manifestations and provide unified mathematical definitions with explicit traceability to the existing literature. Additional anomaly types including meter replacement jumps, data duplication from retransmission, complete missing segments, and timestamp errors are also discussed to present a more complete picture of operational data quality challenges. Second, existing anomaly detection methods are systematically reviewed and classified into four categories—statistical, machine learning, deep learning, and dedicated time-series methods—with representative studies, quantitative performance metrics, and scenario-specific applicability examined for each. Third, data repair approaches are reviewed across four categories—traditional interpolation, matrix completion, generative models, and time-series prediction—with systematic comparison of their accuracy and limitations across different anomaly types and durations. Based on the synthesized evidence, we identify three cross-cutting structural limitations that persist across method categories: the performance ceiling of data-only detection without physical constraint embedding, the open-loop architecture that separates detection from repair and allows error propagation, and the exclusive reliance on statistical error metrics that fails to distinguish physically plausible repairs from those violating conservation laws. To address these gaps, we discuss a physics-guided integrated framework incorporating physical constraint embedding, joint anomaly diagnosis, scenario-adaptive repair, and posterior verification as a promising forward-looking direction. Finally, open challenges and future research directions are outlined, including parameter adaptation in unlabeled scenarios, multi-source data fusion for physical disambiguation, new power system extensions, explainable AI integration, edge-computing deployment, and standardized benchmark development. This review provides a comprehensive theoretical reference and technical roadmap for smart meter data quality research in the context of smart city energy systems.

## 1. Introduction

The construction of smart urban energy systems, characterized by the efficient integration of new energy and the coordinated interaction of urban generation, grid, load, and energy storage, has set unprecedented requirements for the accuracy, real-time performance and integrity of measurement data at the terminal of urban distribution networks [1,2,3,4,5,6]. As the core terminal device for power consumption information collection, smart meters have been widely deployed in numerous cities across China and the globe [7,8,9]. These meters generate massive volumes of electric energy reading data at a 15-min sampling interval (with a 1-min interval adopted in some regions) every day, which serve as the fundamental data support for various smart city energy applications, including urban distribution network state awareness [10], load forecasting [11], demand response, refined line loss management [12,13], electricity theft detection [14,15,16,17,18,19], and accommodation assessment of urban distributed photovoltaic power [20,21].

The value of smart meter data lies not only in the analysis of individual users’ power consumption behaviors, but also in realizing the observability, measurability and controllability of urban distribution networks through data fusion at the transformer district level [22,23,24,25]. For instance, the calculation of line loss in distribution network transformer districts based on smart meter data acts as a core indicator for the “four-classified management” of line loss (by region, voltage level, feeder line and transformer district) implemented by power supply companies, which directly affects line loss assessment and identification of suspected electricity theft. Load forecasting and demand response depend on continuous and accurate user power profiles to facilitate power market clearing and the formulation of orderly power utilization schemes. Data-driven electricity theft detection algorithms identify abnormal power consumption patterns to assist on-site inspections, constituting a vital approach for anti-electricity theft work in cities [26,27,28,29,30]. Accordingly, the quality of smart meter data fundamentally determines the effectiveness and reliability of the aforementioned smart city energy applications.

Nevertheless, smart meters deployed in urban low-voltage distribution networks operate under complex and harsh on-site conditions, and their data quality is threatened by multiple influencing factors [31,32,33,34,35,36,37,38]. Power consumption data acquisition devices in low-voltage transformer districts are continuously exposed to environments with high temperature, high humidity and severe electromagnetic interference. Combined with unstable communication links (including power line carrier, RS485 and public wireless network) [39,40,41], aging metering components inside meters [42], clock drift, man-made electricity theft, and wiring errors, the collected electric energy readings generally suffer from various quality defects. Statistics from a provincial branch of State Grid Corporation of China indicate that a prefecture-level power company of medium scale records tens of thousands of abnormal data entries every day [43], and the abnormal data rate in some transformer districts even exceeds 5%. Such data quality issues severely hinder data-driven analysis and decision-making for distribution networks and may lead to further misjudgments and economic losses [44].

In terms of physical causes and external manifestations, the data quality problems of smart meters mainly include five typical anomalies: sudden jump, reading stagnation, reverse reading, pulse spike, and gradual drift [45,46]. These five types of anomalies differ substantially in data characteristics, duration and physical mechanisms, so a single method cannot effectively address all of them. In recent years, scholars at home and abroad have conducted extensive research on anomaly detection and data recovery technologies for smart meter data and achieved a series of representative outcomes. In terms of anomaly detection, Reference [47] systematically reviews the mainstream technical routes for time-series anomaly detection in smart grids, including statistical methods, machine learning, deep learning and other algorithms. However, this study mainly focuses on performance comparison of different methods, without in-depth discussion on the correlation between physical causes and external manifestations of smart meter data anomalies. Reference [48] proposes a CNN-LSTM method based on cross-layer feature fusion for intrusion detection in advanced metering infrastructure, which achieves satisfactory performance in identifying anomalies from time-series data. Nevertheless, it also fails to analyze the mapping relationship between physical causes and data characteristics of various anomalies. Reference [49] constructs a hybrid deep learning framework for smart meter anomaly detection. Although the detection accuracy is improved, physical constraints of meter data are not incorporated into model design. As for data recovery, Reference [50] presents a data recovery method for user-side meter collected data based on an improved multi-classifier, which can effectively impute missing and abnormal data, yet it does not verify the physical rationality of recovered results. Reference [51] investigates the missing data imputation for power systems and develops a smart meter data interpolation method tailored for energy management systems, which likewise lacks validation on the physical feasibility of recovered data. Reference [52] studies the cleaning and interpolation of power consumption data and realizes data reconstruction via robust non-negative matrix factorization. In this research, anomaly detection and data recovery are treated as two independent processes, and no collaborative optimization mechanism is established be-tween them. Reference [53] explores advanced interpolation approaches for bad and missing smart meter data and puts forward recovery strategies combining statistics and machine learning, but it still cannot realize the collaborative processing of detection and recovery. Reference [54] proposes a reconstruction method for missing distribution network measurement data based on generative adversarial networks and dual semantic perception. Despite its powerful data imputation capability, this method does not systematically embed physical constraints into the recovery process, making it difficult to guarantee the practical applicability of recovered data. Reference [55] develops a missing data imputation method for power grids based on correlation analysis and generative adversarial networks. While it can effectively reconstruct missing data, physical constraints are not integrated to ensure the rationality of recovery results.

To sum up, existing review studies share the following common limitations: (1) a lack of systematic elaboration on the physical causes of anomalies grounded in the specific operational context of urban distribution networks; (2) the separation of anomaly detection and data recovery into independent methodological threads, disregarding their interdependence; (3) insufficient attention to electrical physical laws—such as power monotonicity [56] and energy conservation within transformer districts [57]—as critical prior knowledge differentiating smart meter data from generic time-series data; and (4) research prospects that are not systematically organized around practical smart city deployment requirements.

To address the aforementioned limitations, this paper presents a comprehensive systematic review of anomaly detection and data repair methods for smart meter data. The distinct contributions of this review are as follows:

1. Systematic characterization of five typical anomalies based on physical causation. A causal mapping table linking physical root causes → metering behaviors → data characteristics is established, and rigorous mathematical definitions are formulated for the five types of anomalies with explicit traceability to the existing literature, laying a theoretical foundation for reproducible method comparison.
2. Comprehensive review of anomaly detection methods with quantitative evidence. Existing approaches are classified into four categories: statistical methods, machine learning methods [58], deep learning methods [59], and dedicated time-series methods.
3. Systematic review of data repair methods. Data repair approaches are classified into four groups: traditional interpolation [60], matrix completion [61], generative models [62], and time-series prediction [63]. Common deficiencies in physical interpretability, long-duration repair accuracy, and anomaly-type adaptability are identified.
4. A physics-guided integrated framework for anomaly detection and repair is discussed as a forward-looking direction. This framework integrates physical constraint embedding, joint anomaly diagnosis, scenario-adaptive repair and posterior verification mechanisms, representing a potential solution to the identified gaps in the literature.
5. Open challenges and future research directions are systematically summarized from six perspectives: unsupervised adaptive learning, multi-source data fusion, extension to new power systems, integration of explainable artificial intelligence, edge computing deployment, and standardized test platforms.

This paper consists of seven sections. Section 2 illustrates the fundamentals of smart meter data, as well as the physical causes, data manifestations and mathematical definitions of the five typical anomalies, along with other common anomaly types. Section 3 reviews anomaly detection methods by category and critically analyzes their limitations with quantitative evidence. Section 4 summarizes data repair approaches and their common problems. Section 5 discusses a physics-guided integrated framework as a forward-looking direction. Section 6 outlines open challenges and future research trends. Section 7 draws the final conclusions.

## 2. Characterization of Smart Meter Data and Its Quality Issues

### 2.1. Smart Meter Data Acquisition System

**Figure 1:**
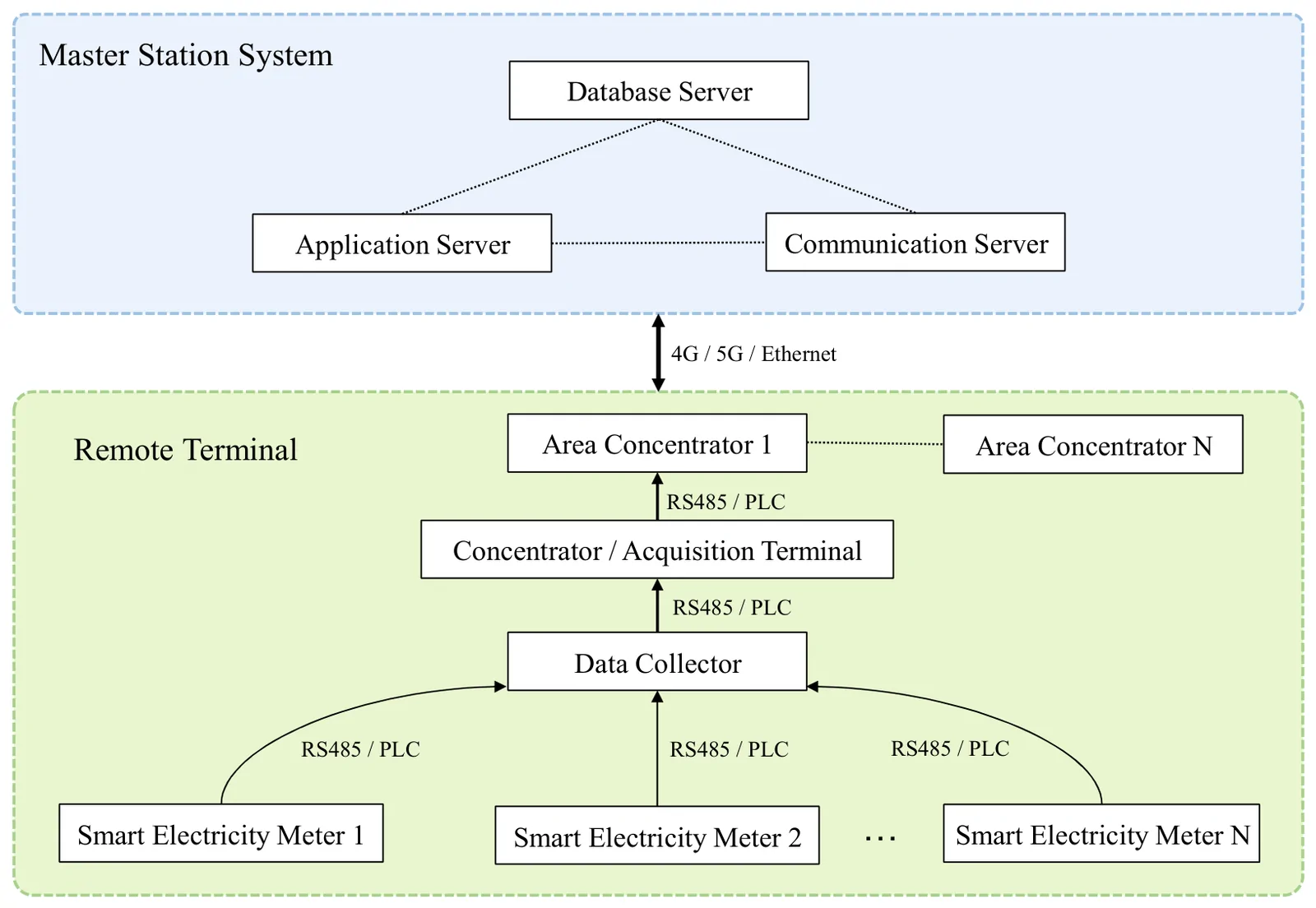
Architecture Diagram of Smart Meter Data Acquisition System in Low-Voltage Distribution Area.

The smart meter data acquisition system serves as the core infrastructure for power consumption information collection in low-voltage distribution networks. Its typical architecture is shown in Figure 1. This system is composed of smart electric meters (sub-meters), concentrators, data collectors [7,64] and a master station system. Smart meters record electrical parameters at fixed sampling intervals (generally 15 min or 1 h), including forward active energy readings, reverse active energy readings, reactive energy, voltage, current and power factor. Among these parameters, the forward active energy reading is the most fundamental and critical data, which is directly applied to user electricity tariff settlement and line loss calculation.

Smart meter data within a low-voltage transformer district are transmitted to the concentrator via Power Line Carrier (PLC) communication, RS485 twisted-pair buses, or RF mesh networks. After data aggregation, the concentrator uploads the data to the master station system through public wireless networks (4G/5G) or wired Ethernet links. Faults occurring at any stage of this multi-hop transmission chain—including malfunctions of internal metering units, communication link interruptions, concentrator buffer overflow, and reception errors at the master station—may all give rise to data quality problems [39,40,43].

Among the local communication technologies, Narrowband PLC (NB-PLC), operating in the CENELEC A band (3–95 kHz) or FCC band (10–490 kHz), has been the dominant solution in smart meter systems for over a decade [22,40]. However, NB-PLC suffers from several inherent limitations: data rates are typically below 100 kbps, and communication reliability is significantly degraded by impulsive noise generated from power electronic devices (e.g., variable frequency drives, switching power supplies) connected to the same low-voltage network. These communication impairments are a major contributor to data quality issues, particularly reading stagnation and data loss [43,44].

In recent years, High-speed Power Line Carrier (HPLC) based on the IEEE 1901.1-2018 standard has been deployed on a large scale by State Grid Corporation of China and China Southern Power Grid [41,65]. HPLC operates in the medium frequency band (<12 MHz) using Orthogonal Frequency Division Multiplexing (OFDM) modulation at the physical layer, achieving data rates of several Mbps with significantly improved robustness against frequency-selective fading and narrowband interference. The media access control (MAC) layer of IEEE 1901.1-2018 employs a centralized scheduling mechanism coordinated by the Central Coordinator (CCO), which manages time slot allocation for each Station (STA, i.e., smart meter) and implements automatic retransmission upon transmission failure. Nevertheless, residual anomalies may still arise from physical layer bit errors during severe channel conditions, MAC layer buffer overflow at the CCO during peak collection periods, and meter-side hardware faults.

Within a low-voltage transformer district, one master meter (gateway metering meter) and multiple sub-meters (user meters) are generally deployed. The master meter records the total electric energy imported from the high-voltage side of the entire district, while each sub-meter measures the power consumption of individual users. In accordance with Kirchhoff’s Current Law, namely the principle of energy conservation, the increment of readings from the master meter is theoretically equal to the sum of increments from all sub-meters when line loss is neglected. In practical operation, the master meter reading increment is slightly larger than the total increment of sub-meters due to inevitable line loss, and the difference corresponds to the line loss of the transformer district. This conservation relation acts as a vital physical constraint for subsequent anomaly detection and data recovery methods.

### 2.2. Mathematical Model of Smart Meter Data

To facilitate subsequent analysis and processing, a mathematical model for smart meter data is established in this section. Suppose there are N users equipped with sub-meters in the transformer district, and the total number of sampling points is T. Let $E_{i}(t)$ (unit: kWh) denote the forward active energy reading of the i-th user at time t, where t=1,2,…,T represents equally spaced sampling instants. The sampling interval Δt is generally 0.25 h (15 min).

The corresponding difference sequence, which represents the average power within each time interval, is defined as

(1) $$p_{i}(t)=E_{i}(t)-E_{i}(t-1),t=2,3,\ldots ,T$$

For unified processing, set $p_{i}(1)=0$. The unit of $p_{i}(t)$ is kWh, indicating the electricity consumption over each 15-min interval. To convert it to the average power in kilowatts (kW), divide the value by Δt (0.25 h), namely $P_{i}(t)=4\cdot p_{i}(t)$. The difference sequence $p_{i}(t)$ is adopted for most of the subsequent analysis in this paper, as it can more intuitively reflect power variations.

For the master meter of the transformer district, its reading is defined as $E_{main}(t)$, and the corresponding difference sequence is $p_{main}(t)$. According to the law of energy conservation, the following equation holds [12,24]:

(2) $$p_{main}(t)=\sum_{i=1}^{N}p_{i}(t)+L(t)$$

where L(t) denotes the line loss energy of the transformer district during time interval t. It generally satisfies $0\leq L(t)\leq \eta_{max}\cdot p_{main}(t)$, in which $\eta_{max}$ stands for the theoretical maximum line loss rate of the transformer district, which is normally no more than 10%.

### 2.3. Physical Characterization and Mathematical Definitions of Typical Anomalies

**Figure 2:**
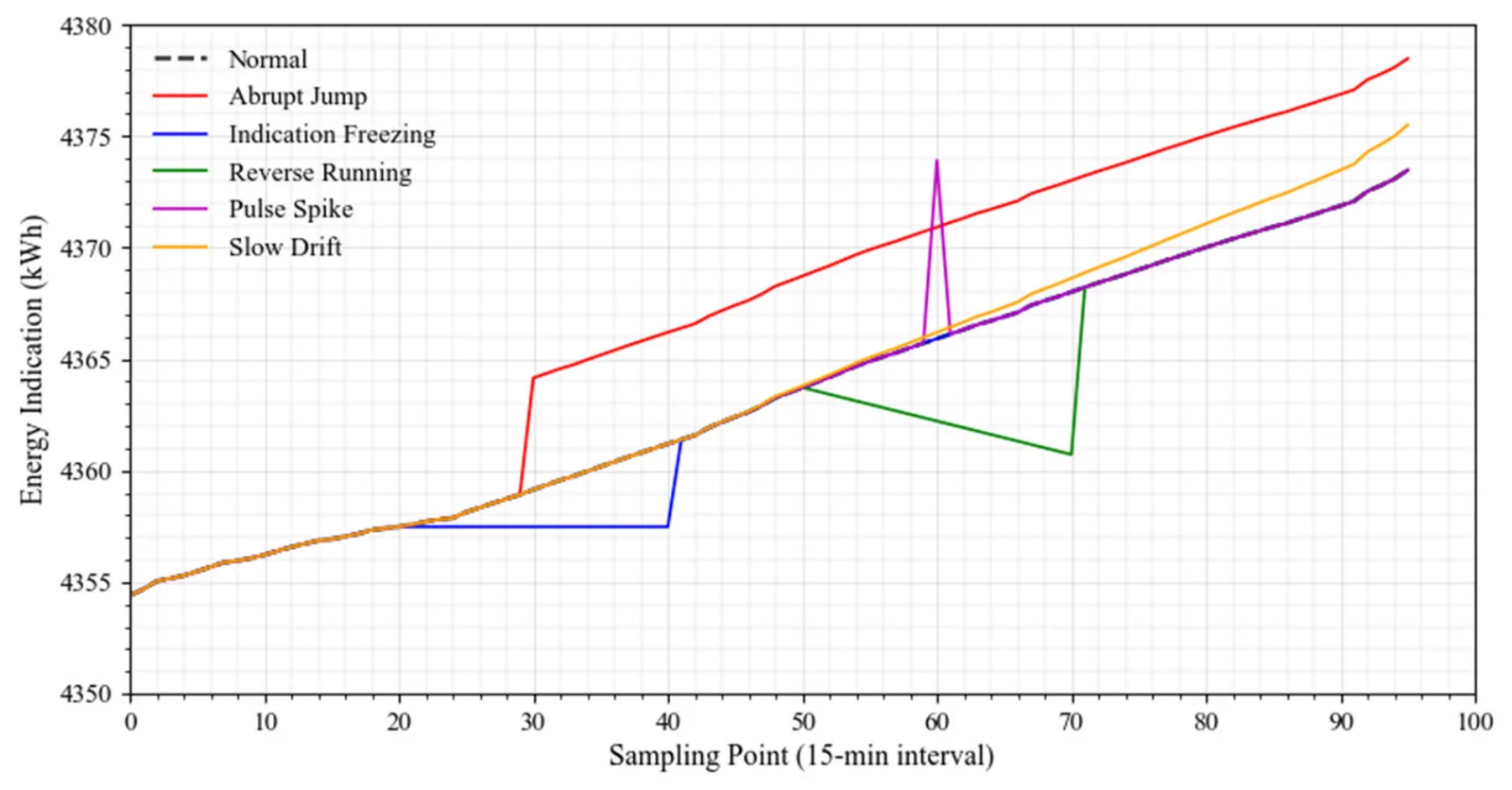
Schematic Diagram of Five Typical Anomalies in Smart Meter Data.

Data quality defects of smart meter readings can be categorized into five typical anomalies, each with distinct physical root causes and corresponding data feature patterns. Table 1 summarizes the physical causes, metering behaviors, data manifestations, and typical duration of the five types of anomalies, and Figure 2 provides schematic illustrations. The qualitative descriptions of these anomaly types have appeared in the existing literature: sudden jumps and abnormal readings are discussed in the context of electricity theft detection and data quality management [44,46]; reading stagnation is recognized as a common consequence of communication link failures [43]; reverse power flow phenomena have been analyzed for distributed photovoltaic users [20]; pulse spikes have been associated with electromagnetic interference in metering circuits [32,46]; and gradual metering drift due to component aging and environmental factors has been documented in [31,34].

To facilitate reproducible detection algorithm design and fair performance comparison, unified rigorous mathematical definitions are presented below. Let $T\in {\mathbb{N}}^{+}$ denote the total number of sampling points. Let the difference sequence be $\Delta P_{t}$ for t=1, 2, …, T. Denote the reference window length as $W\;(W\in {\mathbb{N}}^{+},W<T)$. The mean value and standard deviation of the difference sequence within normal reference windows are *μ* and *σ*, respectively. Define the stagnation discrimination threshold $\varepsilon_{s}$ (generally set to 0.01 kWh or determined adaptively according to noise level) and the reverse reading discrimination threshold $\varepsilon_{r}$ (usually set to 0). All scaling parameters $\left(\alpha_{j},\;\alpha_{s},\;\gamma ,\;\lambda \right)$ are positive real numbers with recommended ranges based on empirical studies.

(1) Sudden Jump

A sudden jump generally occurs when power supply is restored after electricity theft is detected, loose terminal connections re-establish contact, or meter replacement is performed by power utilities, which leads to superposition of readings. When an electricity theft user disables the meter via bypass circuits or rewired metering loops, the meter resumes normal measurement once standard power supply is restored. However, the previously unrecorded electricity volume will not be supplemented, and the meter accumulates readings starting from the current value, resulting in a discontinuous rise. Similarly, abrupt changes in contact resistance caused by loose wiring can also trigger reading jumps.

Under normal operating conditions, the user power curve varies continuously. A sudden jump manifests as a significant deviation in the difference sequence—typically a positive spike exceeding several times the normal fluctuation standard deviation—while the values before and after this instant remain within the normal range. This anomaly usually lasts for only one sampling interval (15 min).

**Definition** **1** (Sudden Jump)**.**

*If there exists a time instant *t∈{2,3,…,T−1}* satisfying*

(3) $$|\Delta P_{t}-\mu |>\alpha_{j}\cdot \sigma$$

*and the difference values at the adjacent instants t − 1 and t + 1 meet the following constraints:*

(4) $$|\Delta P_{t-1}-\mu |\leq \gamma \cdot \sigma ,\;|\Delta P_{t+1}-\mu |\leq \gamma \cdot \sigma$$

*where* $\alpha_{j}$ *is the jump sensitivity parameter (typically ranging from 3 to 5) and *γ *denotes the continuity coefficient (*γ∈[0.5,1.5]*), then t is defined as an abnormal instant of sudden jump [44,46].*

(2) Reading Stagnation

Reading stagnation is mainly caused by communication failures or meter hardware malfunctions. When the communication link is interrupted, the concentrator fails to collect meter readings, and the master station system may fill data with the last valid reading, resulting in pseudo-stagnation. Mechanical jamming of internal counters or faults of electronic metering chips will stop the accumulation of readings, leading to genuine stagnation.

During the stagnation period, the electric energy reading remains constant. Accordingly, the values of the difference sequence stay at or near zero due to measurement noise. The duration depends on the troubleshooting time, ranging from several hours to multiple days.

It should be noted that a near-zero difference sequence is a necessary but not sufficient condition for reading stagnation, because a user may genuinely consume no electricity over an extended period (e.g., an unoccupied household) [66]. In practice, candidate intervals detected by the definition below should be disambiguated using auxiliary contextual information, such as communication status flags from the data concentrator unit and consistency with the user’s historical consumption patterns at the corresponding time of day. These auxiliary signals are further exploited in the physics-guided framework discussed in Section 5.

**Definition** **2** (Reading Stagnation—Necessary Condition)**.**

*If there exists a consecutive subsequence of indices* $n=n_{1},n_{1}+1,\ldots ,n_{2}$*, where* $1\leq n_{1}\leq n_{2}\leq T$ *and* $n_{1},n_{2},T\in {\mathbb{N}}^{+}$*, such that for all n in this range*

(5) $$\left|\Delta P_{n}\right|<\varepsilon_{s}$$

*and the length of the subsequence* $L_{s}=n_{2}-n_{1}+1$* exceeds the stagnation duration threshold* $L_{\min}$* (typically* $L_{\min}\geq 4$*, corresponding to one hour at 15-min sampling), then the subsequence is identified as a candidate stagnation interval requiring further disambiguation [43].*

(3) Reverse Reading

Reverse reading violates the physical principle that the reading of a forward active energy meter is non-decreasing. Its main causes are summarized as follows: (i) incorrect meter wiring with reversed incoming and outgoing current lines; (ii) distributed photovoltaic users without anti-reflux devices—when photovoltaic power generation exceeds local load, surplus power flows back to the grid, and if the meter is not designed for bidirectional measurement, its readings may decrease; and (iii) logical errors in the internal metering direction module of the meter.

Reverse reading is characterized by continuous negative values in the difference sequence, and the magnitude of negative values reflects the reverse power. It should be noted that reverse readings are regarded as normal measurement rather than anomalies for users equipped with bidirectional meters, such as photovoltaic power producers.

**Definition** **3** (Reverse Reading)**.**

*If there exists a consecutive subsequence of indices* $n=n_{1},n_{1}+1,\ldots ,n_{2}$*, where* $1\leq n_{1}\leq n_{2}\leq T$ *and* $n_{1},n_{2},T\in {\mathbb{N}}^{+}$*, such that for all n in this range*

(6) $$\Delta P_{n}<-\varepsilon_{r}$$

*and* $L_{r}=n_{2}-n_{1}+1\geq L_{\min}$*, then the subsequence is identified as reverse reading. This constraint is relaxed for bidirectional-meter users with distributed photovoltaic systems [20].*

(4) Pulse Spike

Pulse spikes are typically induced by transient electromagnetic interference. Lightning surges, startup and shutdown of nearby high-power equipment, and harmonics generated by power electronic devices may produce pulse disturbances during meter sampling and cause abnormal reading fluctuations. In addition, bit flips resulting from channel noise during data transmission can also lead to abnormal individual values.

A pulse spike is an isolated single-point anomaly. The difference sequence shows an extraordinarily high value at one instant, with normal values observed immediately before and after. It differs from a sudden jump: a sudden jump causes a permanent offset and lifts the overall reading level, while a pulse spike stems from transient interference and readings return to the normal level afterwards.

**Definition** **4** (Pulse Spike)**.**

*If there exists a time instant *t∈{2,3,…,T−1} *that satisfies*

(7) $$|\Delta P_{t}-\mu |>\alpha_{s}\cdot \sigma$$

*and additionally*

(8) $$|\Delta P_{t-1}-\mu |\leq \gamma \cdot \sigma ,\;|\Delta P_{t+1}-\mu |\leq \gamma \cdot \sigma$$

*where* $\alpha_{s}$ *is the spike sensitivity parameter (generally set between 4 and 6) and* γ∈(0,1.5] *is the continuity coefficient, then t is defined as an abnormal instant of pulse spike [32,46].*

(5) Gradual Drift

Gradual drift refers to cumulative systematic deviations caused by long-term and gradual faults in the metering process. Typical causes include (i) gradual ratio variation of current transformers (CT) and potential transformers (PT) due to aging or overload, which brings about metering gain errors; (ii) clock drift caused by aging reference voltage sources or crystal oscillators inside the meter, leading to sampling time offset; and (iii) gain drift of metering chips induced by ambient temperature changes.

Gradual drift cannot be detected via single points or short time windows. It is manifested as a remarkable deviation of the mean value of the difference sequence from the historical normal level over a long period, and the deviation accumulates gradually over time. For instance, if a user consumes 10 kWh of electricity per day in actuality, the meter may record 10.5 kWh daily due to ratio errors, forming a notable cumulative deviation over time.

**Definition** **5** (Gradual Drift)**.**

*Let *$L_{a}\in {\mathbb{N}}^{+}$ *denote the length of the long-term window, which is generally set to 96 sampling points (equivalent to one day). The reference window adopts historical normal periods, such as the corresponding time intervals of the previous seven days. The mean value of the difference sequence within the window is defined as*

(9) $$\mu_{d}=\frac{1}{L_{d}}\sum_{k=0}^{L_{d}-1}\Delta P_{t-k}$$

*If there exists a time instant* $t\in \{L_{d},L_{d}+1,\ldots ,T\}$ *satisfying*

(10) $$\frac{\left|\mu_{d}-\mu_{\mathrm{ref}}\right|}{\left|\mu_{\mathrm{ref}}\right|+\varepsilon }>\lambda$$

*where λ stands for the drift rate per unit time and ε is a small positive constant to avoid division by zero, then the window ending at instant t is judged to contain gradual drift anomalies [31,34].*

The threshold parameters involved in the above definitions, including $\alpha_{j},\alpha_{s},\gamma ,\varepsilon_{s},\varepsilon_{r},\lambda$, and $L_{d}$, can be adjusted according to practical application scenarios and noise levels. In field deployment, a sliding reference window can be used to adaptively estimate *μ* and *σ*, thereby reducing sensitivity to global parameters.

While the five anomaly types discussed above represent the most commonly encountered data quality issues in smart meter systems, several additional anomaly types have been documented in power utility field reports and deserve attention:

(1) Meter replacement jumps. When a smart meter is replaced by the power utility due to fault, upgrade, or scheduled rotation, the new meter begins accumulation from zero. This creates a discontinuity in the historical reading sequence that differs from a sudden jump in that the pre- and post-replacement data originate from different physical instruments and no “true value” exists for the transition period. Utility meter management systems typically flag these events through work order records [42].
(2) Data duplication from retransmission. In PLC-based acquisition networks, if the acknowledgment frame from the CCO to the STA is lost due to channel impairment, the STA may retransmit the previously sent data frame. When the CCO’s MAC layer fails to detect the duplicate, the master station receives two identical records for the same timestamp [41].
(3) Complete data missing segments. Distinct from reading stagnation (where the last valid value is repeated), complete missing segments occur when no data frame is received at all for extended periods, typically due to prolonged communication outages or meter power supply failure. The data field is empty rather than filled with a placeholder value, requiring detection at the frame level rather than the value level [40].
(4) Timestamp errors. Clock synchronization errors in meters or concentrators can result in incorrect timestamps that misalign data sequences. When the cumulative clock drift exceeds the sampling interval, measured values are associated with wrong time slots, creating apparent anomalies that are actually temporal misplacements [34].

These additional anomaly types, while less frequent than the five primary categories, represent important edge cases that detection and repair algorithms should be prepared to handle in comprehensive data quality management systems.

## 3. Data Anomaly Detection Methods: Classification, Principles and Limitations

**Figure 3:**
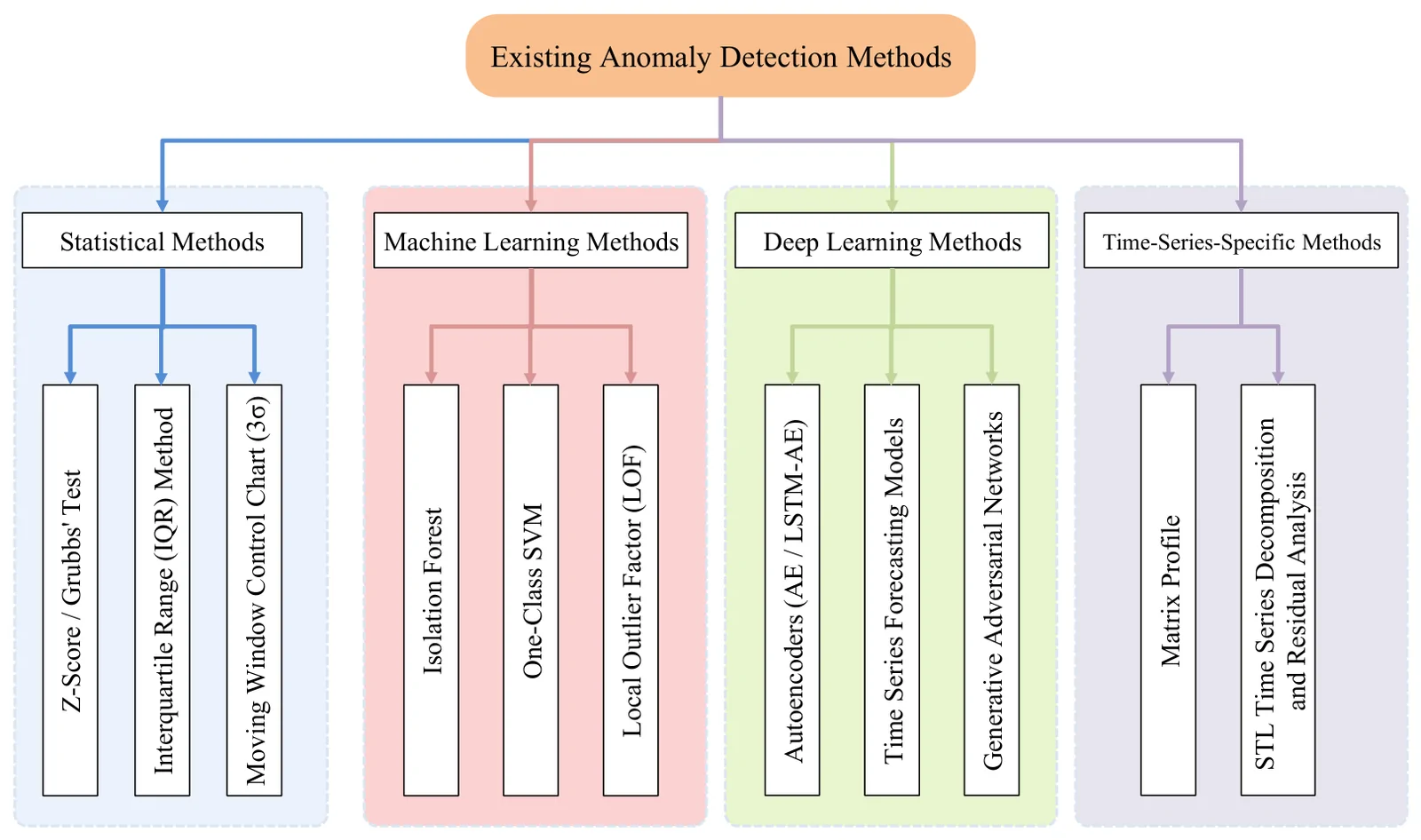
Classification System of Existing Anomaly Detection Methods.

The objective of smart meter data anomaly detection is to identify data points or segments that deviate from normal patterns within time-series data. Based on different technical routes, existing methods can be divided into four categories: statistical methods, machine learning methods, deep learning methods and dedicated time-series methods. Figure 3 illustrates the classification framework and typical representatives of these approaches. This section systematically reviews the core principles, representative studies, applicable scenarios and limitations of each category.

### 3.1. Statistical Methods

Statistical methods are the earliest and most intuitive techniques applied for anomaly detection. Their fundamental assumption is that normal data follow a known probability distribution, while anomalies refer to data points that deviate significantly from this distribution.

#### 3.1.1. Z-Score and Grubbs Test

The Z-score method, also known as the standard score method, identifies anomalies by calculating the deviation multiple of each data point from the sample mean [67]:

(11) $$Z(t)=\frac{\left|p(t)-\mu \right|}{\sigma }$$

A threshold K (generally set between 2.5 and 3.5) is predefined. A data point is classified as anomalous if Z(t)>K. This method features simple computation and easy implementation and is applicable to data that are independent and identically distributed and approximately follow a normal distribution. The Grubbs test [68] is an iterative variant that removes the most extreme point in each iteration and recalculates the mean and standard deviation until no further outliers are detected [69].

In smart meter applications, Jyothi et al. [70] proposed a hybrid framework integrating Z-score with LOF, One-Class SVM, and Isolation Forest, where consensus voting reduces false positives. Krishna et al. [71] systematically compared Z-score, IQR, autoencoder, Isolation Forest, One-Class SVM, and LSTM on smart home energy data, finding that Z-score performs adequately for point anomalies but fails on complex patterns. Mandel et al. [72] applied Z-score to smart water meter data for detecting leaks and abnormal usage patterns. Sleiman [73] utilized Z-score to identify anomalous events at customer-owned PV installations using AMI data. Hela et al. [74] employed Modified Z-Score as a statistical baseline against Time Series Foundation Models on the LEAD 1.0 dataset. For meter verification, Guang Cheng et al. applied the Grubbs test to identify erroneous readings in laboratory and field calibration data [69]. Z-score-based methods achieve acceptable precision (0.85–0.92) for isolated anomalies such as pulse spikes and sudden jumps [70] but exhibit significantly lower recall (<0.60) for stagnation, reverse reading, and gradual drift [74], as stagnation yields near-zero differences, drift shows insignificant single-point deviations, and mean/std estimates are easily distorted by high anomaly proportions [71]. The Grubbs test, while effective for laboratory calibration, suffers from iterative overhead and poor scalability to online deployment [69].

#### 3.1.2. Interquartile Range Method and 3σ Rule

The interquartile range (IQR) method constructs detection intervals based on data quartiles [75,76]. Let $Q_{1}$ denote the first quartile and $Q_{3}$ the third quartile, then $IQR=Q_{3}-Q_{1}$. The normal value range is defined as $\left[Q_{1}-k\cdot IQR,Q_{3}+k\cdot IQR\right]$, where k is generally set to 1.5 for mild anomalies or 3.0 for extreme anomalies. Unlike Z-score, IQR does not assume normality and is more robust to skewed distributions. The 3σ rule assumes that normal data points lie within the interval [μ−3σ,μ+3σ], and any points outside this range are judged as anomalies.

In smart meter applications, Krishna et al. [71] compared IQR with Z-score, autoencoder, and Isolation Forest on smart home energy data, finding IQR effective for point anomalies but limited for complex patterns. Sharma and Tiwari [77] employed IQR with PCA for preprocessing smart grid SCADA data. Hela et al. [74] used IQR as a statistical baseline to benchmark Time Series Foundation Models on the LEAD 1.0 dataset. IQR has also been applied to identify outliers in energy consumption data for tariffing and demand prediction in non-metered environments [78,79]. Despite its robustness, IQR remains a global detection method that ignores temporal dependencies, yielding poor performance for gradual drift and reading stagnation [71,74]. Moving-window 3σ partially addresses non-stationarity but suffers from subjective window selection and insensitivity to drift, as slow variations are averaged out within the window.

#### 3.1.3. Common Limitations of Statistical Methods

Based on the above analysis, statistical methods share several inherent drawbacks when applied to smart meter data anomaly detection, which are summarized in Table 2. These issues fundamentally restrict their detection performance in complex distribution network scenarios. For instance, statistical methods generally perform point-wise detection and cannot fully exploit the contextual information of time series, thereby leading to frequent missed detections of continuous anomalies such as reading stagnation. Moreover, they are highly susceptible to the proportion of anomalous data. Severe data contamination will distort statistical indicators and render detection thresholds invalid. Most importantly, existing statistical methods rarely incorporate electrical physical laws. Their detection results may violate fundamental physical rules including power monotonicity, which makes them inadequate for practical engineering applications.

### 3.2. Machine Learning Methods

Machine learning methods realize anomaly detection by learning normal patterns or classification boundaries from data and are capable of capturing complex nonlinear relationships that statistical methods cannot identify.

#### 3.2.1. Isolation Forest

Isolation Forest (iForest) [80,81] is a tree-based anomaly detection algorithm. Its core principle lies in the fact that anomalies are sparsely distributed in the data space and can be isolated from normal samples via only a small number of random splits. The algorithm constructs multiple random binary trees and defines the anomaly score as the reciprocal of the average path length; shorter paths indicate higher anomaly likelihood. iForest features nearly linear computational complexity, making it suitable for large-scale smart meter data processing.

In smart meter applications, Tambun et al. [79] applied iForest with K-means clustering on Indonesian utility data (2021–2023), achieving 91.54% recall for non-technical loss detection after parameter tuning. Reference [82] proposed a framework combining iForest with FFT filtering for simultaneous time- and frequency-domain detection in distribution networks. Pabbuleti et al. [83] compared iForest and autoencoder on the UCI smart meter dataset, reporting that iForest outperformed autoencoder in both accuracy and recall. Liu et al. [84] developed CWT-iForest integrating continuous wavelet transform with iForest for online single-phase meter error monitoring. Jyothi et al. [70] incorporated iForest into a hybrid voting framework with Z-score, LOF, and One-Class SVM, reducing false positives through consensus voting. Reference [85] proposed improved Canopy-Kmeans combined with iForest for abnormal electricity detection. While iForest achieves competitive precision for isolated anomalies [70,79,82,83], it exhibits three limitations for smart meter data: insensitivity to continuous anomalies such as reading stagnation due to long path lengths [83], disregard for temporal order [82], and high parameter sensitivity [85]. Hybrid approaches with clustering or signal decomposition [82,83,84,85] partially mitigate these issues but add computational overhead.

#### 3.2.2. One-Class Support Vector Machine

One-Class Support Vector Machine (One-Class SVM, OCSVM) [86] maps data into a high-dimensional feature space via kernel functions and seeks a hyperplane that maximizes the separation between most normal data and the origin. Its decision function is expressed as

(12) $$f(x)=sign(\langle \omega ,\phi (x)\rangle -\rho )$$

where ϕ(x) denotes the kernel mapping. OCSVM is applicable to unsupervised scenarios with complex data distributions and an overwhelming majority of normal samples.

For smart meter applications, Wang et al. [87] proposed a Transformer-OCSVM semi-supervised method for electricity theft time identification, validated on smart meter data from a southern Chinese province. A combined Autoencoder-OCSVM framework has been developed for change-and-transmit AMI systems to simultaneously predict consumption and detect theft [88]. For zero-day attack detection, ensemble learning with OCSVM and Gaussian Mixture Models as base learners has been applied to smart meter sensor data [89]. A dedicated OCSVM approach for smart grid energy consumption anomaly detection has been systematically studied [90]. To enhance robustness, an explainable AI-based detector integrating deep autoencoders with OCSVM has been proposed to defend against black-box evasion attacks [91]. Despite its unsupervised learning capability, OCSVM exhibits three limitations for smart meter data: it is sensitive to kernel and regularization parameter selection [87,90]; training complexity scales from $O(n^{2})$ to $O(n^{3})$, limiting large-scale deployment; and it treats time points as independent vectors, discarding temporal order, which degrades performance for continuous anomalies such as stagnation and reverse readings [89]. Accordingly, it yields poor performance on anomalies with distinct temporal patterns, such as reading stagnation and reverse reading.

#### 3.2.3. Common Limitations of Machine Learning Methods

Although machine learning methods relieve the strict data distribution assumptions of statistical methods to some extent, their application to smart meter anomaly detection is still constrained by multiple factors. The common drawbacks are summarized in Table 3. The primary issue is that most machine learning algorithms treat sampling points at different moments as independent and identically distributed samples, thus breaking the inherent temporal dependencies of power consumption data. As a result, these models can hardly identify anomalies with obvious temporal continuity, such as reverse reading and gradual drift. In addition, the anomaly labels generated by machine learning models are difficult to correlate with specific physical root causes, such as communication interruption or wiring errors. When a model trained on one transformer district is transferred to another district with vastly different power consumption patterns, its detection performance usually degrades significantly.

### 3.3. Deep Learning Methods

Deep learning methods construct multi-layer neural networks to automatically extract hierarchical features from data and present prominent advantages in capturing temporal dependencies and complex patterns [92].

#### 3.3.1. Autoencoder and LSTM-Autoencoder

An Autoencoder (AE) consists of an encoder and a decoder trained to minimize reconstruction error on normal data [93]. For anomaly detection, anomalies yield large reconstruction errors, serving as the anomaly score. The LSTM autoencoder (LSTM-AE) integrates LSTM layers [94,95] to encode temporal dependencies [96]. In smart meter applications, a Hybrid CNN-LSTM autoencoder framework [97] achieved 100% precision on a specific cluster for energy theft detection in residential smart meter data. An adaptive LSTM-autoencoder [98] was developed to handle technology-divergent error signals (digital vs. analog) for smart meter metering anomaly detection. Liu et al. [59] proposed V-LSTM (VAE + LSTM) for real-time multi-user electricity metering anomaly monitoring, validated on both public and proprietary datasets. An LSTM-LSTM autoencoder [99] outperformed baseline methods for detecting anomalies in commercial building load profiles. On the UCI smart meter dataset, reference [83] compared autoencoder with Isolation Forest for energy theft identification, reporting that autoencoder achieved competitive performance but required substantially more training data. While autoencoder-based methods achieve state-of-the-art sensitivity for isolated anomalies [97,99], they require large volumes of clean normal data for training [59], suffer from trivial reconstruction of stagnation patterns [98], and lack physical constraint embedding [83].

#### 3.3.2. GAN-Based Methods

Generative Adversarial Network (GAN)-based anomaly detection methods generally follow the normal-sample generation plus discriminator-classification paradigm [100]. The generator learns the manifold of normal consumption patterns, while the discriminator distinguishes real from generated samples [101,102]. In smart meter applications, a conditional GAN (CGAN) framework [103] has been developed for electricity theft detection, leveraging adversarial learning to reveal hidden patterns in consumption data. The TRANS-DEEP framework [104], an optimized deep learning approach, achieved detection accuracies of 94.66%, 96.34%, and 95.12% across three smart meter datasets (SMD-I, II, III). For improved interpretability, a semi-supervised GAN combined with generalized additive neural networks [105] has been proposed for smart city electricity theft detection. Despite promising accuracy, GAN-based detection suffers from unstable training, non-unique latent variable inference, high computational cost, and physically infeasible reconstructions that violate power monotonicity and rated capacity limits, hindering edge deployment.

#### 3.3.3. Core Advantages and Common Limitations of Deep Learning Methods

Capable of automatically extracting hierarchical temporal features, deep learning methods achieve high sensitivity in detecting isolated anomalies including sudden jumps and pulse spikes. Even so, they still face considerable challenges in practical engineering deployment. Their core advantages and common limitations are summarized in Table 4. Specifically, deep learning methods impose strict requirements on both the quality and quantity of training data. Since perfectly clean normal data are hardly obtainable in actual transformer districts, models tend to be biased by contaminated samples. Meanwhile, the black-box nature of deep models leads to poor physical interpretability, making it difficult for operation and maintenance staff to understand the model decisions. Furthermore, these models show weak generalization across different districts or seasons and incur high retraining costs. They rarely incorporate electrical constraints such as power conservation and monotonicity and may produce physically unreasonable results.

### 3.4. Dedicated Time-Series Methods

Dedicated time-series methods are specially designed for the characteristics of time-series data, commonly combining techniques such as time series decomposition, similarity measurement and subsequence analysis.

#### 3.4.1. Matrix Profile

Matrix Profile (MP) [106] is a time series analysis framework that efficiently computes the distance between each subsequence and its nearest neighbor. Anomalous subsequences are identified as those with the greatest distances to all other subsequences, i.e., the most dissimilar patterns. MP has a time complexity of O(*n^2^*) for naive computation, accelerated to O(*n* log *n*) using Fast Fourier Transform and the MASS algorithm. In smart meter applications, reference [107] applied MP to power measurement data from advanced smart meters, demonstrating its relative robustness for anomaly detection in electricity consumption profiles. Reference [108] proposed an unsupervised theft detection framework combining MP with K-means clustering, achieving effective identification of abnormal consumption patterns without labeled data. For industrial customers, the Sleuth model [109] integrated MP with LSTM networks for time-series pattern recognition and anomaly detection in power consumption behavior. MP has also been tested on photovoltaic active power measurement datasets for online embedded analytics in LVDC microgrids [110]. While MP effectively detects short-term abnormal patterns such as sudden jumps and pulse spikes [107,110], it exhibits three limitations for smart meter data: poor performance on reading stagnation, where repetitive zero sequences yield small mutual distances and escape detection [108]; insensitivity to gradual drift, as slowly varying subsequences remain similar to their lagged counterparts [109]; and high sensitivity to subsequence length parameter, which must match the target anomaly duration [110].

#### 3.4.2. STL Decomposition and Residual Analysis

STL (Seasonal-Trend decomposition using Loess) is a robust time series decomposition technique. It decomposes a series into trend component T(t), seasonal component S(t) and residual component R(t):

(13) X(t)=T(t)+S(t)+R(t)

For smart meter data with strong daily periodicity, statistical methods such as Z-score or IQR can be applied to the residual component for anomaly detection, effectively eliminating interference from trends and seasonal patterns. In smart grid applications, Zhang et al. [111] applied STL decomposition to power grid data and detected anomalies by applying statistical methods to the residual component. A cloud computing-based approach [112] employed STL to decompose smart meter data on a cloud platform for scalable anomaly analysis. More recently, an improved STL-Loess algorithm integrated with dynamic thresholding has been proposed for hybrid anomaly detection and correction in modern power systems [113]. STL provides clear physical interpretability: the trend reflects long-term consumption variations, the seasonal component captures daily periodic behaviors, and the residual represents random fluctuations [111]. However, it has two limitations for smart meter data: poor adaptability to irregular periodicities caused by holidays or unexpected events [112], and sensitivity to bandwidth parameters for trend and seasonal windows [113].

#### 3.4.3. Common Limitations of Dedicated Time-Series Methods

By explicitly modeling seasonality, trends and subsequence similarity of time series, dedicated time-series methods provide a new perspective for anomaly detection different from the above approaches. Nevertheless, they still have inherent drawbacks when applied to smart meter data, as summarized in Table 5. A typical problem is that most methods are highly targeted for specific anomaly types. For example, Matrix Profile nearly fails to detect repetitive patterns like reading stagnation, while STL decomposition produces large deviations in seasonal component estimation when confronted with irregular cycles such as holidays. In addition, these methods are generally sensitive to parameters including subsequence length and decomposition window size. The optimal parameters vary across different users and time periods, resulting in heavy manual tuning workload. Although these methods better capture temporal characteristics than conventional machine learning methods, they still lack the integration of physical constraints.

### 3.5. Summary of Common Deficiencies of Existing Anomaly Detection Methods

Several comprehensive surveys have systematically reviewed anomaly detection techniques for smart grid and smart meter data [47,49]. Zhang et al. [47] provided a systematic overview of mainstream technical routes for time-series anomaly detection in smart grids, covering statistical methods, machine learning, deep learning, and other algorithms. Kaur et al. [49] proposed a novel hybrid deep learning framework for intelligent smart meter anomaly detection, demonstrating state-of-the-art performance through extensive experimentation. The general shortcomings of current anomaly detection techniques applied to smart meter data are summarized as shown in Table 6.

(1) Separation of detection and recovery. Most detection methods operate as standalone modules that output binary flags without characterizing anomaly type, duration, or contextual reliability—information essential for downstream repair. Conversely, repair outcomes are not fed back to refine detection, resulting in an open-loop architecture that permits error propagation.
(2) Lack of physical interpretability. Statistical, machine learning and deep learning methods mostly identify anomalies purely from the perspective of data distribution, without explicitly embedding electrical physical laws including power monotonicity, district energy conservation and power upper limit. This leads to two problems: firstly, false judgments may occur. For instance, normal reverse power flow of photovoltaic users may be misclassified as reverse reading anomalies. Secondly, detection results can hardly be linked to specific physical root causes, which hinders field operation and maintenance staff from quickly locating faults.
(3) Inadequate capability for continuous and trending anomalies. Anomalies such as reading stagnation (continuous zero values), reverse reading (continuous negative values) and gradual drift (long-term mean offset) do not present obvious abnormal features at single data points. Their identification relies on continuous contextual information or statistics from long windows. Existing methods, especially conventional machine learning approaches, generally perform poorly on such anomalies.
(4) Limited cross-scenario generalization ability. Parameters or models trained for a specific transformer district or season suffer from severe performance degradation when migrated to other user groups or time periods. Deep learning models require retraining with high computational costs, while statistical and time-series methods need manual threshold adjustment due to the absence of adaptive mechanisms.
(5) Heavy parameter dependence and insufficient adaptive configuration. Nearly all methods rely on multiple threshold parameters, such as Z-score threshold, contamination rate of Isolation Forest, reconstruction error threshold for LSTM, subsequence length of Matrix Profile and window size of STL. These parameters are usually set empirically or optimized offline with labeled datasets and cannot be adjusted adaptively in practical unlabeled deployment scenarios.
(6) Neglect of district-level topological information. Most methods conduct detection independently for individual user time series and fail to exploit correlations among multiple users as well as the energy conservation constraint between master meters and sub-meters within a transformer district. In fact, metering anomalies of a single user will generate identifiable signals in the residual of district line loss, which can be leveraged to improve detection reliability.

**Table 6:** Comparative Summary of Anomaly Detection Method Categories.

Dimension	Statistical	Machine Learning	Deep Learning	Dedicated Time-Series
Representative Algorithms	Z-score, IQR, 3σ, Grubbs test	iForest, OCSVM, LOF	AE, LSTM-AE, GAN, Prediction-based	Matrix Profile, STL
Best-suited Anomaly Types	Isolated spikes, severe jumps	Moderate outliers in high-dim. spaces	Jumps, spikes (with sufficient data)	Short-term pattern deviations
Weakest Anomaly Types	Stagnation, drift, reverse readings	Stagnation, drift (context-insensitive)	Stagnation (trivial reconstruction)	Stagnation (repetitive patterns)
Training Required	No	Yes (unsupervised)	Yes (large-scale)	No (parameter-dependent)
Physical Constraint Embedding	None	None	None	None
Cross-Scenario Generalization	Requires threshold tuning	Moderate; degrades with pattern shift	Poor; requires retraining	Requires window/season tuning
Interpretability	High (statistical rationale)	Moderate to low	Very low (black-box)	Moderate (component interpretable)

## 4. Data Repair Methods: Classification, Principles and Limitations

Data repair aims to correct or fill identified abnormal data points and segments, so as to restore data integrity and accuracy. Different from general time-series missing value imputation, smart meter data repair faces two distinctive challenges: (1) various types of anomalies require differentiated repair strategies; (2) repaired data must comply with electrical physical rules. This chapter reviews existing data repair methods with an emphasis on published quantitative results and cross-method comparisons.

### 4.1. Traditional Interpolation and Statistical Methods

The methods reviewed in this section—including traditional interpolation techniques, which are among the most fundamental and widely adopted approaches for data repair—share a common premise: they rely primarily on the local or periodic structure of the time series itself, without requiring external training data. Traditional interpolation methods estimate abnormal values based on the mathematical relationships of valid data in the neighborhood, a characteristic that makes them computationally efficient and easily deployable on edge devices, which is why they remain the most widely adopted approaches in operational utility systems. However, as the subsequent analysis will show, these methods exhibit systematic performance degradation when applied to long-duration anomalies or physically constrained scenarios.

#### 4.1.1. Linear Interpolation and Spline Interpolation

Linear interpolation estimates abnormal values by assuming linear changes between adjacent valid points, while spline interpolation fits piecewise polynomials for smoother results. Both methods feature low computational complexity and no training requirement, making them widely deployed in operational utility systems. For AMI systems, a hybrid method combining linear interpolation with LSTM estimates has been proposed for missing data compensation [114]. In a comprehensive study of 3021 smart heat meters in Danish residential buildings, linear interpolation was applied to handle timestamp deviations, with weighted moving average selected after systematic comparison of nine imputation methods [115]. Peppanen et al. [53] conducted a comprehensive evaluation of imputation methods for bad and missing smart meter data, concluding that linear interpolation is suitable for short missing periods (<4 h) but exhibits rapid error accumulation for longer gaps. The compiled evidence reveals a clear pattern: interpolation methods achieve acceptable RMSE only for short-duration anomalies (<4 h) [53,115]; for long-duration stagnation (>6 h), errors accumulate rapidly [114]. Furthermore, no study verifies physical feasibility (e.g., district energy balance) of interpolation results, confirming the gap identified in Section 5.

#### 4.1.2. Historical Mean Imputation and Periodic Mean Imputation

Historical mean imputation adopts the average power value of the same time slot over previous days for the corresponding user as the repaired value:

(14) $$\hat{p}(t)=\frac{1}{D}\sum_{d=1}^{D}p_{d}(\tau )$$

where τ denotes the time index within a day (e.g., the τ-th 15-min sampling point), and D is the number of historical days.

Periodic mean imputation typically uses a 7-day or 30-day reference period to leverage daily and weekly consumption cycles. In residential smart meter data, a benchmarking study [116] evaluated periodic mean imputation alongside other load forecasting methods, concluding that it performs well for regular consumption patterns with strong periodicity. Peppanen et al. [53] comprehensively assessed historical mean imputation for bad and missing smart meter data, confirming its effectiveness for short-term gaps under stable consumption behavior. Despite its simplicity and prior knowledge utilization, periodic mean imputation produces large errors for non-periodic consumption caused by holidays or abrupt behavioral changes and fails when historical data at the same time slot are also anomalous [53,116]. Simple averaging also tends to smooth out intraday fluctuations, distorting the variation characteristics of the original load profile [53].

#### 4.1.3. Multiple Imputation

Multiple Imputation (MI) [117] generates multiple repaired datasets by repeatedly sampling from the predictive distribution. The final repaired value is obtained by synthesizing statistical results such as mean and variance from these versions, and the uncertainty of repair is quantified simultaneously. Common implementations include regression-based methods and the Markov Chain Monte Carlo (MCMC) method.

This method, which provides confidence intervals for repaired values and is applicable to scenarios requiring uncertainty quantification (e.g., risk analysis), has limitations: high computational complexity rendering it unsuitable for large-scale real-time data repair, reliance on assumptions about the missing mechanism (typically missing at random), which may not hold for smart meter anomalies belonging to missing not at random, and failure to enforce physical constraints.

#### 4.1.4. Common Limitations of Traditional Interpolation Methods

Thanks to low computation cost and easy deployment, traditional interpolation methods are widely adopted for preliminary data repair in practical engineering. Nevertheless, they present prominent drawbacks when applied to complex anomalies of smart meter data, which are summarized in Table 7. The most critical issue lies in long-duration anomalies, such as reading stagnation lasting for several hours or even days. Linear or spline interpolation merely using valid values adjacent to the abnormal interval leads to rapidly accumulated extrapolation errors along with the extension of abnormal segments, rendering the repaired data practically useless. Furthermore, these methods adopt a uniform interpolation strategy for all anomaly types, without designing dedicated operators for anomalies with different physical causes, such as sudden jumps and reverse readings. More importantly, the repaired results have no physical constraints guaranteed and may easily produce negative power values or readings exceeding the rated capacity.

### 4.2. Matrix Completion-Based Methods

Matrix completion methods represent a paradigm shift from local interpolation to global structure exploitation. Instead of relying on temporal proximity, these methods leverage correlations across users—a natural property of power consumption data within the same transformer district—by organizing the dataset into a user-time matrix, where rows represent users and columns denote time instants, and recovering missing or abnormal entries through the low-rank property of this matrix. This section reviews the principles, applications, and limitations of matrix completion for smart meter data repair, with emphasis on the conditions under which the low-rank assumption holds and fails.

Matrix completion methods leverage the low-rank property of the user–time consumption matrix, where rows represent users and columns denote time instants, to recover missing or abnormal entries. The recovery is formulated as a nuclear norm minimization problem, solvable via Singular Value Thresholding (SVT) or ADMM. In distribution system state estimation, matrix completion has demonstrated superior performance over linear interpolation on the IEEE 37-bus system [118]. However, studies emphasize that system equations must be incorporated as constraints to improve estimation accuracy [119]. For theoretical line loss calculation in low-voltage distribution networks, SVT-based matrix completion has been introduced to obtain high-precision data [120]. A three-step power quality data restoration system comprising preprocessing, matrix completion, and result validation has been proposed for the power Internet of Things [121]. Robust nonnegative matrix factorization has also been applied to electricity consumption data cleansing and imputation [52]. Matrix completion performs well under three conditions: high user homogeneity, low anomaly proportion (<10%), and approximately low-rank structure. However, it has three limitations for smart meter data: the low-rank assumption fails in heterogeneous transformer districts mixing residential, commercial, and industrial users [118,120]; prior knowledge of anomaly locations is required [121]; and computational complexity of SVD and iterative optimization is O(*n*^3^), hindering real-time deployment [119]. Improved variants—clustering with block-wise completion, time-series regularized completion, and graph-regularized completion—partially address these issues but still lack physical constraint embedding and introduce additional parameter tuning complexity [52]. Reference [122] adopts low-rank matrix completion to recover missing power load data, achieving better performance than traditional interpolation methods. Reference [123] applies matrix completion to restore grid voltage measurement data and effectively improves data fitting under low-observation conditions.

### 4.3. Generative Model-Based Methods

Generative models approach data repair from a distributional perspective: rather than interpolating or predicting, they learn the high-dimensional probability distribution of normal power consumption patterns and generate samples conforming to that distribution, conditioned on observed data. Representative approaches include Generative Adversarial Networks (GANs) and Variational Autoencoders (VAEs). This section reviews these methods, comparing their theoretical advantages—such as global consistency and long-gap imputation—with practical limitations, including training requirements, computational costs, and physical feasibility concerns. The recent emergence of diffusion models is also discussed as a promising frontier.

#### 4.3.1. GAN-Based Repair Methods

Generative models approach data repair from a distributional perspective, learning the high-dimensional probability distribution of normal power consumption patterns and generating samples conditioned on observed data [124]. Reference [125] adopts Wasserstein GAN (WGAN) to enhance training stability and achieves higher repair accuracy than matrix completion under high missing rates. For repair tasks, a GAN consists of a generator G and a discriminator D, where the generator is first trained on normal data to produce realistic power consumption curves. Given observed data x containing anomalies, the latent variable z is optimized so that G(z) best matches the observed parts, and the values at anomalous positions of G(z) are taken as the repaired results [124]. In smart meter applications, the GAN-Transformer framework [126] integrates GANs with Transformer encoders to jointly model temporal dynamics and feature correlations for multivariate power consumption imputation. SAGANConvLSTM [127] combines semivariogram-enhanced GAN with ConvLSTM for spatio-temporal power load forecasting with missing value imputation. CC-GAIN [128] proposes a clustering and classification-based generative adversarial imputation network specifically for missing electricity consumption data. An improved BAC-GAN model [129] has been developed for missing data recovery in power systems. Design of experiments for Pix2Pix hyperparameter analysis has been systematically studied for energy consumption data imputation [130]. The classical GAN-based multivariate time series imputation framework by Luo et al. [124] provides the foundational methodology for this paradigm. Compared with traditional interpolation, GAN-based methods maintain global distribution consistency and capture complex temporal dependencies automatically, performing well for long missing segments [126,127]. However, they suffer from unstable training, mode collapse, high computational cost, time-consuming latent variable optimization, and lack of physical feasibility guarantees [128,129]. Recent diffusion models have emerged as a promising alternative but remain largely unexplored for smart meter repair [130].

#### 4.3.2. Common Limitations of Generative Model-Based Methods

By learning the high-dimensional data distribution, generative models, especially GANs and VAEs, are theoretically capable of producing repaired values that conform to normal power consumption patterns and perform well for repairing long missing segments. Even so, they still face inevitable challenges in practical engineering applications, which are summarized in Table 8. Firstly, these models require a large volume of high-quality and fully normal data for training. In practice, data collected from transformer districts are often contaminated by various anomalies, making it hard to obtain pure training datasets. Secondly, generative models incur extremely high computational costs. Both training and inference require multiple iterative optimizations, which prevents real-time operation on edge devices such as data concentrators. Most importantly, the end-to-end black-box generation process cannot guarantee that repaired results comply with physical rules including power monotonicity and district energy conservation. In addition, it is difficult to trace and rectify unreasonable repair outputs.

### 4.4. Time-Series Prediction-Based Methods

Time-series prediction methods approach data repair from a forecasting perspective: for a detected anomaly at time t, the value is replaced by a prediction generated from preceding observations. These methods build forecasting models using historical data and fill abnormal intervals with predicted values, relying on extrapolation based on recent historical observations rather than learning a global data distribution—unlike generative models. This paradigm is naturally suited to online repair scenarios where data arrive sequentially. This section reviews classical ARIMA and Holt–Winters models alongside deep learning-based predictors such as LSTM and TCN, analyzing their error propagation characteristics and the critical distinction between one-step and multi-step prediction for long-duration anomaly repair.

#### 4.4.1. Classical Time Series Models

The ARIMA (Auto Regressive Integrated Moving Average) model [131] formulates time series as a linear combination of autoregressive (AR) and moving average (MA) components. For abnormal points, one-step or multi-step predictions generated by ARIMA using past observations are adopted as repaired values.

The Holt–Winters exponential smoothing method [132] updates recursively via level, trend, and seasonal components and is suitable for data with trends and periodicity. In smart meter applications, its triple exponential smoothing variant can capture daily cycles and weekend effects. These models feature few parameters, fast computation and good interpretability. However, they have three major drawbacks. First, the linear assumption limits their capacity to fit nonlinear power consumption patterns. Second, prediction errors accumulate rapidly for long-term forecasting; the longer the reading stagnation lasts, the less reliable the extrapolated results become. Third, model orders need manual selection, and the methods cannot adapt automatically to different anomaly types.

#### 4.4.2. LSTM-Based Prediction and Repair

Time-series prediction-based repair methods construct forecasting models from historical data and replace abnormal points with predicted values. For a detected anomaly at time *t*, the value is replaced by a prediction generated from preceding observations. This paradigm is naturally suited to online repair scenarios where data arrive sequentially. Classical ARIMA and Holt–Winters models formulate time series as linear combinations of autoregressive, moving average, and seasonal components, featuring few parameters and good interpretability. LSTM networks mitigate the vanishing gradient problem via gating mechanisms and excel at capturing long-range dependencies in complex power consumption patterns. In AMI systems, a comparative study [133] evaluated GRU, LSTM, and SARIMA for 15-min interval load data imputation, concluding that LSTM and GRU significantly outperform SARIMA for missing data estimation. A dedicated investigation [134] of LSTM methods for AMI missing data estimation confirmed their superiority in capturing complex temporal patterns. For distribution system state estimation, a Transformer-Bi-LSTM imputation model [135] has been proposed for missing measurements. In field deployment, LSTM-based basic error prediction models have been constructed and deployed on smart meter on-site operation data [136]. Despite strong nonlinear fitting capability, LSTM-based repair suffers from strict requirements for large quantities of clean training data [134]; rapid error accumulation for multi-step prediction on continuous anomalies such as stagnation [133,135]; absence of physical constraints; and lack of differentiation among anomaly types—optimal strategies vary for jumps, spikes, and drift, while a single prediction model cannot adaptively switch [136].

#### 4.4.3. Common Limitations of Time-Series Prediction-Based Methods

Time-series prediction-based repair methods construct forecasting models from historical data and fill abnormal points or segments with predicted values, delivering acceptable performance for single-point anomalies and short-term missing data. Nevertheless, their common drawbacks in smart meter data repair are summarized in Table 9. The major weakness lies in long-duration anomalies such as reading stagnation lasting several hours. Multi-step extrapolation relying on predicted values causes rapid accumulation and amplification of errors, making the repaired curves deviate drastically from actual power consumption trends.

In addition, these methods adopt a one-size-fits-all strategy and apply the same prediction mechanism to all anomaly types. They fail to design targeted correction operations for the offset characteristics of sudden jumps and the reverse features of reverse readings. Strict requirements for clean training data and the lack of physical constraints further restrict their reliable application in complex transformer districts.

### 4.5. Summary of Common Deficiencies of Existing Data Repair Methods

Based on the review of the four categories of methods above, the general shortcomings of current data repair techniques for smart meter data are concluded as follows:

(1) Disconnection between detection and repair. Most methods separate anomaly detection and data repair into two independent stages. Errors including false positives, missed detections and misclassification of anomaly types are directly passed to the repair module with no feedback mechanism. For instance, interpolating normal points mistakenly labeled as anomalies introduces extra errors. If sudden jumps are misidentified as spikes, inappropriate repair operators (interpolation instead of bias correction) will be adopted, leading to physically inconsistent repaired data.
(2) Lack of physical feasibility. This is the most critical issue. Existing methods generally take minimizing Mean Squared Error (MSE) as the optimization objective, without considering whether the repaired data comply with electrical physical rules such as power monotonicity, district energy conservation and power upper limits. Consequently, although the repaired data achieve favorable statistical error indicators, they may produce unreasonable results when applied to line loss calculation or electricity theft detection, such as negative line loss rates.
(3) Failure to distinguish anomaly types. Different anomalies including sudden jumps, reading stagnation, reverse readings, spikes and gradual drift have distinct physical mechanisms and data characteristics. An ideal repair scheme should design dedicated operators for each anomaly type. However, most existing methods adopt a one-size-fits-all strategy such as interpolation, prediction or generation. They cannot perform bias correction for sudden jumps, historical curve reconstruction for stagnation, or signal inversion for reverse readings.
(4) Low accuracy for long-duration anomaly repair. The repair error of all conventional methods rises sharply when anomalies last for several hours (stagnation) or even days (drift). Long abnormal segments provide few valid neighboring normal data, and the extrapolation capability of prediction and generative models is inherently limited.
(5) Strong parameter dependence and poor adaptability. Parameters such as the order of ARIMA, window size and network layers of LSTM, rank of matrix completion, and latent dimension of GAN require scenario-specific tuning, and model performance is highly sensitive to these settings. In practical deployment, optimal parameters vary among individual users, making manual tuning impractical.
(6) High computation and deployment costs. Deep learning models such as LSTM and GAN require time-consuming training and inference. Matrix completion relies on iterative optimization. These approaches are difficult to run in real time on data concentrators and edge devices with limited computing resources [137].

## 5. Discussion

The systematic review in Section 3 and Section 4 surveyed about 100 publications spanning statistical, machine learning, deep learning, and time-series-dedicated approaches for anomaly detection and data repair. When these methods are examined not in isolation but as a collective body of evidence, several convergent patterns emerge. This section synthesizes three cross-cutting findings that, together, outline a coherent research frontier for smart meter data quality.

### 5.1. The Limits of Data-Only Detection

The quantitative evidence assembled in Section 3 tells a consistent story: no existing detection approach achieves satisfactory performance across all five anomaly types simultaneously. Systematic evaluations show that stagnation and drift detection remain the primary failure modes across all ML architectures [47]. Deep learning methods push the ceiling higher—F1 up to 0.94 for jumps and spikes [49,96]—but at the cost of requiring tens of thousands of clean training samples per user, a condition rarely met in operational transformer districts [47].

A structural pattern underlies these performance ceilings. All four method categories share a common premise: anomalies are defined as statistical deviations from a learned or assumed distribution of normality. None of them exploit the physical knowledge that smart meter data are generated by circuits obeying Kirchhoff’s laws, that transformer district energy must be conserved [57], or that each anomaly type has a specific physical cause traceable to metering hardware or communication infrastructure (Table 1, Section 2.3). The recognition that physical context is not merely auxiliary but constitutive of anomaly definition has emerged independently in adjacent domains—physics-informed machine learning for power system applications [138], or causal graph-guided fault diagnosis in industrial systems—but has not yet been systematically applied to smart meter data quality. Building an explicit physical causal model that constrains detection logic from first principles represents a logical next step substantiated by the literature’s collective limitations.

### 5.2. The Detection–Repair Divide and How to Close It

A second structural pattern emerges from the review of repair methods in Section 4. With near-universal consistency, existing approaches—whether interpolation-based [52,54], matrix completion [61,106,122], generative [54,55,62], or prediction-based [51,63]—treat the anomaly mask as a pre-existing input. In operational deployments, however, this mask is generated by an upstream detector, and any detection error propagates directly into the repair output. The consequences are quantifiable: recent studies have shown that repair RMSE can increase substantially when the gap boundary is misidentified [50]. Conversely, none of the detection studies reviewed in Section 3 evaluate detection performance in terms of downstream repair quality. The literature thus exhibits a one-way, open-loop architecture that existing surveys have noted without proposing a structural remedy [47,53,54,55].

Closing this loop requires two interdependent changes. First, detection must output not a binary anomaly flag but a richer characterization—anomaly type, temporal extent, and an estimate of contextual reliability—that the repair stage can use to select and parameterize an appropriate correction strategy. Second, repair outcomes must serve as feedback: a repaired segment that violates a well-established physical law (e.g., the transformer district energy balance) constitutes evidence that the original detection label was incorrect, warranting re-evaluation. This bidirectional perception–action–verification architecture has not been explored in the smart meter data quality literature. A corollary finding concerns repair strategy selection: the uniform-strategy problem documented in Section 4—where the same logic is applied regardless of anomaly type—is a direct consequence of the open-loop architecture, since a binary flag provides no basis for differentiating among anomaly mechanisms that require fundamentally different correction logic.

### 5.3. Physical Feasibility: The Missing Evaluation Criterion

The third convergent finding concerns evaluation standards. Every detection study reviewed in Section 3 reports F1-score, recall, and precision. Every repair study in Section 4 reports MSE, MAE, and R^2^. Not a single study reports whether the repaired data satisfy basic electrical conservation laws. When GAN-based imputation produces physically infeasible values [62,95], or when interpolation of multi-day stagnation yields large values [50,52], the statistical error metrics alone are misleadingly optimistic—a district with a negative calculated line-loss rate triggers false electricity-theft investigations, and a repaired load curve exceeding the user’s rated capacity corrupts transformer loading assessments.

The physical principles themselves are not new. Monotonicity of metering accumulation has been recognized since electronic meters were introduced. Energy conservation in transformer districts is a direct consequence of Kirchhoff’s Current Law and has been employed as a consistency check in state estimation [57]. Continuity-based outlier rejection predates smart meters, appearing in early SCADA data quality work [67,68]. What the literature lacks is the unified formalization of these scattered constraints into a verifiable, computable constraint set that can serve as a standardized quality gate for any repair method. The development of such a constraint set, accompanied by physical feasibility metrics in benchmark evaluations, would close a fundamental gap between academic method development and utility engineering requirements.

### 5.4. Summary

The reviewed literature collectively supports three converging priorities: physics-embedding addresses the performance ceiling of data-only detection by incorporating domain knowledge into the detection logic; integration addresses the open-loop architecture by establishing bidirectional information flow between detection and repair; and physical feasibility verification addresses the evaluation gap by making constraint compliance a mandatory quality gate. Each of these priorities is grounded in specific, quantifiable deficiencies documented across Section 3 and Section 4. Realizing them in operational systems requires progress on a number of concrete technical challenges, examined in Section 6.

## 6. Open Challenges and Future Research Directions

The findings synthesized in Section 5 point to clear priorities but depend on resolving several open challenges for their translation into practice. This section focuses on those challenges most directly connected to the core themes of this review—anomaly detection and data repair methodology—and the evaluation infrastructure needed to advance them.

### 6.1. Methodological Challenges

#### 6.1.1. Parameter Adaptation in Unlabeled Scenarios

The physically informed detection paradigm discussed in Section 5.1 depends on reliable estimates of normal-behavior statistics and anomaly-sensitivity parameters. Acquiring these estimates is challenging because labeled anomaly data are scarce—utility field records capture only the small fraction of anomalies that trigger customer complaints or billing exceptions [43]—and because normal consumption baselines drift over time due to seasonal shifts, appliance adoption, and occupancy changes [139]. Three complementary approaches merit investigation: unsupervised and self-supervised learning to extract normal data representations without labels [138]; online adaptive threshold adjustment using sliding-window statistics with automatic window-length adaptation [140]; and Bayesian change point detection to distinguish genuine anomalies from legitimate consumption regime shifts [141].

#### 6.1.2. Multi-Source Data Fusion for Physical Disambiguation

The stagnation-versus-zero-load ambiguity (Section 2.3) demonstrates that single-source detection using only active energy readings cannot resolve physically distinct scenarios. Smart meters measure voltage, current, power factor, reactive power, and frequency—quantities linked by the power triangle P^2^ + Q^2^ = S^2^ and the proportionality of current to power at constant voltage—which provide independent evidence channels [142,143,144]. When active power is near zero, yet voltage is present and current is near zero, the meter records genuine non-consumption; when voltage data are missing or current channels exhibit anomalies, communication failure is the more probable cause. Formalizing such cross-channel consistency rules, and extending them through multi-modal fusion architectures [145,146,147,148] and graph-structured topological priors that exploit the electrical proximity of meters within a transformer district [149,150], would directly address this ambiguity.

#### 6.1.3. Hierarchical Detection for Mixed Anomalies

The five-anomaly taxonomy adopted in this review—and in nearly all published detection studies—treats anomaly types as mutually exclusive. Operational data, however, frequently contain compound anomalies: a stagnation interval may embed a transient pulse spike, a gradual drift may be punctuated by sudden jumps, or a reverse reading segment may be superimposed on slow ratio degradation. The current single-label paradigm is structurally incapable of modeling such compositions. Two directions show promise: multi-pass sequential diagnosis—short-duration detectors applied first, long-duration detectors on the residual—with careful management of error propagation across passes [151,152], and multi-scale window ensembles that fuse parallel detection outputs from single-point to weekly windows through confidence-weighted voting [153,154]. Neither has been validated on smart meter-specific anomaly mixtures, and the construction of labeled datasets with per-data-point ground truth for compound anomaly scenarios is a prerequisite for progress.

### 6.2. Ecosystem Challenges

#### 6.2.1. Adapting to New Power System Scenarios

The accelerating deployment of distributed photovoltaics, EV charging, and battery storage alters the operational assumptions underlying current detection and repair methods [155]. Bidirectional power flow breaks the monotonicity assumption; EV charging pulses resemble spike anomalies; and storage charge–discharge cycles mimic reverse readings [156,157]. The core challenge is adaptive constraint management—migrating from static physical constraints to context-dependent rules, with IEC 62053-series standards providing the normative framework [158]. At the district level, automated topology identification from voltage correlation analysis [159,160] is needed to keep energy conservation baselines synchronized with dynamic meter list changes.

#### 6.2.2. Standardized Benchmarks and Evaluation Protocols

The absence of standardized benchmarks is arguably the single largest barrier to reproducible progress. Three priorities stand out: (1) a public benchmark dataset spanning diverse user categories and at least one full annual cycle, with expert-verified per-data-point labels and auxiliary metadata (communication logs, meter replacement records, PV installation status); (2) an open-source anomaly injection toolkit with controllable parameters for amplitude, duration, contamination ratio, and mixed-anomaly composition; and (3) a multi-dimensional evaluation framework that adds physical feasibility metrics (constraint violation rate, post-repair line-loss error) and per-anomaly-type breakdowns to the traditional F1/MSE/MAE metrics.

## 7. Conclusions

Smart meter data is a core asset for urban distribution network governance. Its quality directly determines the reliability of load forecasting, line loss management, demand response, and electricity theft prevention. This paper has presented a systematic review of anomaly detection and data repair methods for smart meter data, covering over 150 publications. The major findings are summarized as follows.

(1) Anomaly characterization grounded in physical causation (Section 2). Five typical anomalies—sudden jumps, reading stagnation, reverse readings, pulse spikes, and gradual drifts—have been systematically characterized through a complete causal mapping chain from physical root causes to data manifestations. Rigorous mathematical definitions with corrected notation have been formulated, building on qualitative descriptions from the existing literature and providing a unified foundation for reproducible method comparison. Additional anomaly types (meter replacement jumps, data duplication, complete missing segments, and timestamp errors) and the stagnation-versus-zero-load ambiguity have been discussed to present a more complete picture of operational data quality challenges.
(2) Systematic review of detection and repair methods (Section 3 and Section 4). Detection methods have been classified into four categories (statistical, machine learning, deep learning, and dedicated time-series) and systematically reviewed through representative studies. The consistent finding is that no single method handles all five anomaly types adequately: statistical methods fail on stagnation, machine learning methods are insensitive to temporal context, and deep learning methods demand clean training data and sacrifice interpretability. Data repair approaches—interpolation, matrix completion, generative models, and time-series prediction—share two cross-category deficiencies: repair accuracy degrades sharply with anomaly duration in all method families, and no method provides a guarantee of physical feasibility. These deficiencies propagate directly into downstream calculations such as line loss assessment and electricity theft detection.
(3) Cross-cutting synthesis of the reviewed literature (Section 5). Beyond method-specific limitations, this review identifies three cross-cutting patterns that recur across method categories yet remain unaddressed. Physical knowledge—that metering circuits obey Kirchhoff’s laws and each anomaly type has a specific physical genesis—remains external to all existing pipelines. The universal separation of detection from repair creates an open-loop architecture in which detection errors propagate unchecked into repair outputs. And the exclusive reliance on statistical error metrics for evaluation fails to distinguish physically plausible repairs from those that violate conservation laws. Identifying and documenting these cross-cutting patterns—visible only when the literature is examined collectively—constitutes the principal meta-contribution of this review. Each pattern points to a research priority substantiated by convergent evidence from independent studies.
(4) Open challenges and research roadmap (Section 6). Key open challenges directly connected to the core themes of detection and repair have been identified: parameter adaptation in unlabeled and non-stationary scenarios; multi-source data fusion for physical disambiguation of ambiguous anomaly patterns; hierarchical detection for compound anomalies; adaptation to bidirectional metering, EV charging, and battery storage in new power systems; and the construction of standardized benchmark datasets with expert-verified anomaly labels and multi-dimensional evaluation frameworks incorporating physical feasibility metrics.

Looking ahead, smart meter data quality research is evolving from statistical deviation-based detection toward physically informed, causally interpretable data governance. The cross-cutting patterns documented in this review—the performance limits of data-only detection, the propagation of errors through decoupled pipelines, and the evaluation gap left by statistical metrics alone—collectively highlight where the field’s attention should concentrate. As edge computing and urban digital twin infrastructures mature, a new generation of data governance technologies will achieve the transition from passive anomaly response to active data resilience, providing a reliable data foundation for smart city energy systems.

## Figures and Tables

**Table 1:** Physical Causes and Data Manifestations of Five Typical Anomalies.

Anomaly Type	Physical Root Causes	Metering Behavior	Data Manifestation	Typical Duration
Sudden Jump	Power restoration after electricity theft, reconnection after loose wiring, abrupt change in metering loop impedance	Instantaneous reading jump	Isolated large positive values in the difference sequence	Single point
Reading Stagnation	Communication link interruption, short-term failure of acquisition equipment, meter counter stuck	Reading stops accumulating	Differences are zero or nearly zero at consecutive sampling points	Several hours to days
Reverse Reading	Wiring errors (reversed polarity), reverse current (photovoltaic users without anti-reflux protection)	Reading continuously decreases	Consecutive negative values in the difference sequence	Several hours to days
Pulse Spike	Lightning surges, harmonic interference, bit flips during data transmission	Transient abnormal reading jump followed by recovery	Isolated single point with extremely large difference, normal before and after	Single point to several points
Gradual Drift	Long-term deviation of transformer ratio, aging of metering components, clock drift	Systematic deviation accumulation in readings	Mean value of differences shifts over a long time window	Several days to months

**Table 2:** Common Limitations of Statistical Methods for Data Anomaly Detection.

Limitation	Explanation
Lack of temporal context	Statistical methods typically perform point-wise detection and fail to leverage the correlation and trends between adjacent data points.
Weak detection capability for continuous anomalies	The Z-score of reading stagnation (consecutive zero values) may not be high; the single-point deviation of gradual drift may be very small.
Sensitive to the proportion of anomalies	When anomalies account for a high proportion of data, the mean and standard deviation are severely contaminated, leading to inaccurate detection thresholds.
Absence of physical constraints	Electrical physical laws such as power monotonicity and district energy conservation are not incorporated, which may lead to physically unreasonable detection results.

**Table 3:** Common Limitations of Machine Learning Methods for Data Anomaly Detection.

Limitation	Explanation
Loss of temporal information	Most machine learning methods treat each time point as an independent sample, which destroys the inherent temporal dependence of power consumption data.
Weak detection capability for continuous/trending anomalies	Anomalies with continuous or trending characteristics, such as reading stagnation, reverse readings, and gradual drift, are difficult to be effectively identified by isolated point detection methods.
Lack of physical interpretability	Detection results cannot be mapped to specific physical root causes (e.g., communication interruptions, wiring errors), which is not conducive to on-site fault troubleshooting.
Limited cross-scenario generalization capability	The performance of a model trained on one transformer district drops significantly when migrated to another district with different power consumption patterns.

**Table 4:** Core Advantages and Common Limitations of Deep Learning Methods for Data Anomaly Detection.

Problem	Explanation
High data dependency	Requires a large amount of normal data for training; it is difficult to obtain a clean training set in practical scenarios.
“Black-box” nature	Lacks physical interpretability; it is difficult to trace detection results back to specific physical root causes.
Poor cross-scenario generalization	Significant differences in consumption patterns among different districts and seasons require retraining the model.
High training cost	Deep learning models are time-consuming to train, have high computing power requirements, and are difficult to deploy on lightweight edge devices.
Lack of physical constraints	Electrical physical laws such as monotonicity and conservation are not embedded, which may lead to physically impossible outputs.

**Table 5:** Common Limitations of Dedicated Time-Series Methods for Data Anomaly Detection.

Limitation	Explanation
Overly specific to anomaly types	Matrix profile fails to detect stagnation; STL is sensitive to irregular periodicity, making it difficult to handle all five types of anomalies uniformly.
High parameter sensitivity	Parameters such as subsequence length and decomposition window need to be adjusted for different users or time periods.
Relatively high computational cost	Online decomposition updates or Matrix Profile (MP) require considerable computing resources.
Persistent lack of physical constraints	Electrical physical laws are not used to distinguish true anomalies from normal fluctuations.

**Table 7:** Common Limitations of Traditional Interpolation Methods for Data Repair.

Limitation	Explanation
Low accuracy in repairing long-duration anomalies	For anomalies with long durations such as stagnation and drift, estimation errors accumulate when relying solely on neighboring normal values.
Lack of physical constraints	Repaired data may exhibit physically unreasonable results, such as negative power, values exceeding rated capacity, or violations of energy conservation.
Failure to leverage concurrent/periodic information (linear/spline)	Relies only on local temporal context, ignoring the strong prior of daily periodicity.
No differentiation between anomaly types	All anomalies use the same interpolation strategy, with no dedicated repair operators designed for distinct anomaly mechanisms such as jumps, spikes, and reverse readings.

**Table 8:** Common Limitations of Generative Model-Based Methods for Data Repair.

Limitation	Explanation
Requires large amounts of complete normal data for training	It is difficult to ensure that the training data is completely clean in practical transformer districts.
Physical constraints are not embedded	Generated results may violate monotonicity and energy conservation laws.
High computational cost	Training is time-consuming, and the inference phase also requires multiple forward propagations or iterative optimization.
Poor interpretability	The repair process is a black box, which is not conducive to engineering auditing and fault tracing.
Uncertainty in repairing long-duration anomalies	The longer the anomaly persists, the lower the credibility of the generated results.

**Table 9:** Common Limitations of Time Series Prediction-Based Methods for Data Repair.

Limitation	Explanation
Large extrapolation error for long-duration anomalies	The longer the stagnation or drift lasts, the larger the deviation of predicted values.
High purity requirement for training data	If the training set contains anomalies, the model will be contaminated.
Lack of anomaly type adaptability	The same prediction mechanism is used for all anomalies, with no dedicated operators designed for jumps (offset correction) or reverse readings (inversion).
Insufficient physical constraint guarantees	Predicted values may exceed physically allowable ranges.

## Data Availability

No new data were created or analyzed in this study. Data sharing is not applicable to this article.

## References

[1] Yang J., Wang C., Wang Z. (2025). Review of intelligent load forecasting algorithms in the context of new power systems. J. North China Electr. Power Univ. (Nat. Sci. Ed.).

[2] Pau M., Patti E., Barbierato L., Estebsari A., Pons E., Ponci F., Monti A. (2018). A cloud-based smart metering infrastructure for distribution grid services and automation. Sustain. Energy Grids Netw..

[3] Wang Y., Zhong Q., Liu Y., Tu C., Zhang H. (2025). Review and prospects of key technologies for power quality disturbance mitigation in new-type power systems. Proc. CSEE.

[4] Li P., Zu W., Liu Y., Tian C., Hao Y., Li H. (2025). State estimation method for distribution network based on incomplete measurement data. Electr. Power.

[5] Han X., Li T., Zhang D., Zhou X. (2021). New issues and key technologies of new power system planning under double carbon goals. High Volt. Eng..

[6] Hu J., Cao D., Hu W., Chen J., Chen Z. (2023). Robust state estimation method for distribution network based on graph neural network incorporating topology knowledge. Autom. Electr. Power Syst..

[7] Dong Y., Li X., Yu J., Zhang L., Bai L. (2023). Data acquisition architecture and implementation of new smart electricity meter based on optical fiber communication. Electr. Meas. Instrum..

[8] (2016). Technical Administrative Code of Electric Energy Metering, DL/T 448-2016. https://www.ndls.org.cn/standard/detail/c515ab1c3415673a72df4ab1dd65bc95.

[9] Ye R., Yan X., Li T. (2026). Optimization and application of smart meter data acquisition technology. China Sci. Technol. J. Database Ind. C..

[10] Chen L., Lao K.-W., Ma Y., Zhang Z. (2022). Error modeling and anomaly detection of smart electricity meter using TSVD plus+L method. IEEE Trans. Instrum. Meas..

[11] Wang S., Deng X., Chen H., Shi Q., Xu D. (2021). A bottom-up short-term residential load forecasting approach based on appliance characteristic analysis and multi-task learning. Electr. Power Syst. Res..

[12] Lyu Y., Chen W., Cheng Y., Su Y., Chen F., Liu X. (2025). Joint estimation method of smart meter operating error and daily line loss rate based on constrained optimization model. Power Syst. Technol..

[13] Song X., Zhang J., Cui C., Huang L., Luo Y., Zeng X. (2024). A new method for diagnosing abnormal line loss in low-voltage transformer areas based on electricity consumption information collection data. Electr. Meas. Instrum..

[14] Jin S., Su S., Xue Y., Yang Y., Liu S., Cao Y. (2022). Review on data-driven based electricity theft detection method and research prospect for low false positive rate. Autom. Electr. Power Syst..

[15] Madhure R.U., Raman R., Singh S.K. CNN-LSTM based Electricity Theft Detector in Advanced Metering Infrastructure. Proceedings of the 2020 11th International Conference on Computing, Communication and Networking Technologies (ICCCNT).

[16] Liu Y., Zha Z., Jiang Y. (2022). Analysis of anti-theft and violation by current method in low-voltage station area. Instrum. Equip..

[17] McLaughlin S., Podkuiko D., McDaniel P. (2010). Energy Theft in the Advanced Metering Infrastructure. Critical Information Infrastructures Security (CRITIS 2009).

[18] Chen Q., Zheng K., Kang C., Huangfu F. (2018). Detection methods of abnormal power consumption: Review and prospect. Autom. Electr. Power Syst..

[19] Huang Q., Yang M., Wang W., Liu Y., Chen H., Jin T. (2026). A novel two-stage self-attention weight fusion model for missing data imputation in electricity theft detection. Power Syst. Technol..

[20] Qiu J., Li H., Wang Z., Lan D., Zhang Y., Liu L. (2023). Research and application of three-phase line loss theoretical calculation method for low-voltage transformer areas with distributed photovoltaics. Distrib. Util..

[21] Caballero-Peña J., Cadena-Zarate C., Parrado-Duque A., Osma-Pinto G. (2022). Distributed energy resources on distribution networks: A systematic review of modelling, simulation, metrics, and impacts. Int. J. Electr. Power Energy Syst..

[22] Gungor V.C., Sahin D., Kocak T., Ergut S., Buccella C., Cecati C., Hancke G.P. (2013). A survey on smart grid potential applications and communication requirements. IEEE Trans. Ind. Inform..

[23] Dudek G. (2016). Pattern-based local linear regression models for short-term load forecasting. Electr. Power Syst. Res..

[24] Wei X., Gao W., Yang G. (2022). Smart meter error estimation method based on improved dynamic line loss estimation. Electr. Technol..

[25] Chen Z. (2022). Line loss calculation and loss reduction measures analysis of low-voltage distribution network. Lamps Light..

[26] Xia X., Xiao Y., Liang W., Cui J. (2022). Detection methods in smart meters for electricity thefts: A survey. Proc. IEEE.

[27] Lakshmi G., Gopal R. Design and Development of IoT Prototype for Real-Time Theft Detection and Optimization of Electricity Using Machine Learning Techniques. Proceedings of the 2024 2nd International Conference on Computing and Data Analytics (ICCDA).

[28] Mihaela Buzau M., Tejedor-Aguilera J., Cruz-Romero P., Gomez-Exposito A. (2019). Detection of non-technical losses using smart meter data and supervised learning. IEEE Trans. Smart Grid.

[29] Glauner P., Meira J.A., Valtchev P., State R., Bettinger F. (2017). The challenge of non-technical loss detection using artificial intelligence: A survey. Int. J. Comput. Intell. Syst..

[30] Kolade A.O., Adetokun B.B., Oghorada O. Energy theft detection in power system network: Reviews of studies on machine learning based solutions. Proceedings of the 2023 International Conference on Science, Engineering and Business for Sustainable Development Goals (SEB-SDG).

[31] Xiong S., Zhang J., Zhang B., Sun G., Chen Z., Qi J., Sun Y. (2021). Effects of environmental and electrical factors on metering error and consistency of smart electricity meters. Appl. Sci..

[32] Leferink F., Keyer C., Melentjev A. (2016). Static energy meter errors caused by conducted electromagnetic interference. IEEE Electromagn. Compat. Mag..

[33] Kuwalek P., Wiczynski G. (2021). Dependence of voltage fluctuation severity on clipped sinewave distortion of voltage. IEEE Trans. Instrum. Meas..

[34] Zhu Y., Jin Y., Tao L., Bao Z., Gong G., Zhou Y. (2023). Research on prediction of clock battery undervoltage for smart electricity meter based on system life distribution. Electr. Meas. Instrum..

[35] Zhang B., Xia X., He C., Kang W., Zhang J. (2024). Examining performance calibration in smart power system electricity metering based on environmental perception attention LSTM-network. Front. Energy Res..

[36] Ji S., Shu Q. (2025). Research on abnormal detection of user power metering based on carrier technology. Electr. Eng..

[37] Wang X., Li X., Xie J., Wang C., Zhang Y. (2025). Failure test design and implementation for electromagnetic disturbance of digital circuits in smart electricity meters. J. Sichuan Univ. (Nat. Sci. Ed.).

[38] Peng L. (2025). Research on abnormal data identification and processing technology of electricity information acquisition system in rural power marketing. Consum. Electron..

[39] Rao R., Akella S., Guley G. Power Line Carrier (PLC) Signal Analysis of Smart Meters for Outlier Detection. Proceedings of the 2011 IEEE International Conference on Smart Grid Communications (SmartGridComm).

[40] Omar M.S., Naqvi S.A.R., Kabir S.H., Hassan S.A. (2017). An experimental evaluation of a cooperative communication-based smart metering data acquisition system. IEEE Trans. Ind. Inform..

[41] Sendin A., Losada-Sanisidro P., Garcia-Rodriguez J., Gonzalez-Mendez P., Berganza I. (2024). Broadband over power line communication prototype development for next generation smart meters: Validation in access electric power distribution networks. IEEE Access.

[42] Xiang J. (2023). Intelligent electric energy metering collection system operation, maintenance and fault treatment analysis. Electron. Technol..

[43] Xiang Y. (2023). Study on influencing factors of low-voltage acquisition success rate for power consumers. Rural Electrif..

[44] Huang G., Fu T., Lin S., Dong P., Xue B. (2023). Research on abnormal identification of electric energy metering data in electricity consumption information collection system. Power Syst. Clean. Energy.

[45] Zheng A., Zhang M., Qu M., Zhao B., Chen H., Xiong Q. (2018). Prediction of fault types of smart electricity meters based on Bayesian network. Electr. Meas. Instrum..

[46] Wu H., Jiang G. (2017). Detection and analysis of power data based on quantum filter. Telecommun. Sci..

[47] Zhang J., Wu D., Boulet B. Time series anomaly detection for smart grids: A survey. Proceedings of the 2021 IEEE Electrical Power and Energy Conference (EPEC).

[48] Yao R., Wang N., Liu Z., Chen P., Sheng X. (2021). Intrusion detection system in the advanced metering infrastructure: A cross-layer feature-fusion CNN-LSTM-based approach. Sensors.

[49] Kaur S., Chowhan P., Sharma A. (2025). A novel hybrid deep learning-based framework for intelligent anomaly detection in smart meters. IEEE Access.

[50] Tang D., Li Y., He W., Liu Y., Ou Y., Wu L. (2023). Restoration method for user electricity meter collection data based on improved multiple classifiers. Autom. Electr. Power Syst..

[51] Wang M.-C., Tsai C.-F., Lin W.-C. (2021). Towards missing electric power data imputation for energy management systems. Expert Syst. Appl..

[52] Liu Q., Zhong Y., Lin C., Li T., Yang C., Fu Z., Li X. (2024). Electricity consumption data cleansing and imputation based on robust nonnegative matrix factorization. Power Syst. Technol..

[53] Peppanen J., Zhang X., Grijalva S., Reno M.J. Handling Bad or Missing Smart Meter Data through Advanced Data Imputation. Proceedings of the 2016 IEEE Power & Energy Society Innovative Smart Grid Technologies Conference (ISGT).

[54] Yang Y., Qi L., Wang H., Su L., Xu Y. (2020). Missing data reconstruction method for distribution network measurement data based on generative adversarial network and dual semantic awareness. Autom. Electr. Power Syst..

[55] Cai R., Yang X., Tian J., Zhao Q., Wang Y. (2024). A power system missing data filling method based on correlation analysis and generative adversarial network. Electr. Power Eng. Technol..

[56] Yang D., Wang Y., Li Z., Gong X., Yu J., Li L. (2022). Forecasting-aided state estimation of integrated energy systems based on improved particle filter. Electr. Power Eng. Technol..

[57] Zhang X., He G., Li K., Zhong M., Liu K. (2022). Hybrid state estimation method for electric-thermal integrated energy systems. J. Electr. Power Sci. Technol..

[58] González C.M., Galvis J., Rosero-Garcia J. (2026). Detection of anomalies in electricity consumption patterns using density-based clustering: A hybrid PCA-HDBSCAN approach applied to advanced metering data. Appl. Sci..

[59] Liu K., Jia X., Wang J., Ma X., Li J. (2025). Real-time monitoring and simulation of multi-user electricity metering anomaly data based on distributed system. IEEE Access.

[60] Pang X. (2012). A comparative study of imputation methods for missing data. Stat. Decis..

[61] Jiang R., Yu X., Jin X., Jia H., Qi F., Mu Y., He B. (2025). Spatio-temporal measurement data completion method for distribution network based on alternating minimization matrix completion and moving average. Electr. Power Autom. Equip..

[62] Wang S., Chen H., Pan Z., Wang J. (2019). A reconstruction method for missing data in power system measurement using improved generative adversarial network. Proc. CSEE.

[63] Zhang D., Li W., Liu Y., Liu C. (2014). Reconstruction method for abnormal operating data of active power in wind farms. Autom. Electr. Power Syst..

[64] Wu L. (2014). Research on the design of intelligent electric energy collection system of distribution power grid. China Electr. Power (Technol. Ed.).

[65] (2018). IEEE Standard for Power Line Communication—Medium Frequency (Less Than 12 MHz) Power Line Communication Transceivers.

[66] Aligholian A., Farajollahi M., Mohsenian-Rad H. Unsupervised learning for online abnormality detection in smart meter data. Proceedings of the 2019 IEEE Power & Energy Society General Meeting (PESGM).

[67] Leys C., Ley C., Klein O., Bernard P., Licata L. (2013). Detecting outliers: Do not use standard deviation around the mean, use absolute deviation around the median. J. Exp. Soc. Psychol..

[68] Grubbs F.E. (1969). Procedures for detecting outlying observations in samples. Technometrics.

[69] Xie G., Chen W., Liu X., Zheng K., Zou B., Sun H. Research and application of verification error data processing of electricity meter based on Grubbs criterion. Proceedings of the 2019 IEEE 3rd Information Technology, Networking, Electronic and Automation Control Conference (ITNEC).

[70] Jyothi B., Pabbuleti B., Mounika B., Kalapala H., Chandra M.U.S., Srilakshmi S.S., Babu B.G., Rao K.V.G., Kumar M.K., Chilakala R.R. (2026). Empowering energy management: Anomaly detection in smart meter data for proactive consumption control. Bull. Electr. Eng. Inform..

[71] Krishna N.S., Kumar Y.P., Prakash K.P., Reddy G.P. (2024). Machine learning and statistical techniques for outlier detection in smart home energy consumption. 2024 IEEE Open Conference of Electrical, Electronic and Information Sciences (eStream).

[72] Kolla T., Kurian A., Babu T. (2025). Big data analytics for urban water consumption using smart meter data. 2025 3rd International Conference on Advances in Computation, Communication and Information Technology (ICAICCIT).

[73] Sleiman A. (2024). Identified BTM Customer Owned PV Anomalies by Considering Load Profiles, Voltage Data and Load Forecasts Using AMI, Customer and Weather Data.

[74] Hela B., Handigol P.P., Arjunan P. Are time series foundation models good for energy anomaly detection?. Proceedings of the 16th ACM International Conference on Future and Sustainable Energy Systems.

[75] Hyndman R., Fan Y. (1996). Sample quantiles in statistical packages. Am. Stat..

[76] Ma L., Geng Y., Yuan N., Duan X. (2023). Abnormal data cleaning method for wind turbines based on quartile and CFSFDP. Electr. Power Sci. Eng..

[77] Sharma A., Tiwari R. (2024). Anomaly detection in smart grid using optimized extreme gradient boosting with SCADA system. Electr. Power Syst. Res..

[78] Muyulema-Masaquiza D., Ayala-Chauvin M. (2025). Segmentation of energy consumption using K-means: Applications in tariffing, outlier detection, and demand prediction in non-smart metering systems. Energies.

[79] Tambun R., Priyadi A., Lystianingrum V. (2025). Anomaly detection of non-technical losses in smart meter data using K-means clustering and isolation forest: A case study from Indonesia. 2025 International Seminar on Intelligent Technology and Its Applications (ISITIA).

[80] Cheng Y., Li F., Yin J., Xu X., Zhu L., Ying X. (2026). Anomaly monitoring of electricity consumption in chain power grid structure based on isolation forest. Inf. Technol..

[81] Tong Z., Niu Z. (2026). Intelligent detection method for abnormal values in power big data based on isolation forest algorithm. Electr. Eng..

[82] Labura P., Antić T., Capuder T. (2025). Time and frequency domain-based anomaly detection in smart meter data for distribution network studies. 2025 IEEE Kiel PowerTech.

[83] Elzubair M.J., Musa R.M., Eldeen R.M.A.T., Elbashir R.E., Alkhalifa T.A., Saeed R.A., Saeed M.M., Elbasheir M.S., A Mokhtar R. (2026). Unsupervised anomaly detection for energy theft identification in smart grids using isolation forest and autoencoder models. 2026 IEEE 5th International Maghreb Meeting of the Conference on Sciences and Techniques of Automatic Control and Computer Engineering (MI-STA).

[84] Liu B. (2026). On-line monitoring of single-phase smart meter operation error based on wavelet transform and machine learning. AIP Adv..

[85] Wang J., Li X. (2024). Abnormal electricity detection of users based on improved canopy-kmeans and isolation forest algorithms. IEEE Access.

[86] Schölkopf B., Platt J.C., Shawe-Taylor J., Smola A.J., Williamson R.C. (2001). Estimating the support of a high-dimensional distribution. Neural Comput..

[87] Chen J., Wang M., Liu Y., Jiang H., Miao X., Lin W., Zheng C., Zhao R. (2025). Electricity theft time identification method based on Transformer and one-class support vector machine. Power Syst. Technol..

[88] Bondok A., Abdelsalam O., Badr M., Mahmoud M., Alsabaan M., Alsaqhan M., Ibrahem M.I. (2024). Accurate power consumption predictor and one-class electricity theft detector for smart grid ‘change-and-transmit’ advanced metering infrastructure. Appl. Sci..

[89] Massarani A.H., Badr M.M., Baza M., Alshahrani H., Alshehri A. (2025). Efficient and accurate zero-day electricity theft detection from smart meter sensor data using prototype and ensemble learning. Sensors.

[90] Agbo E., Olajide I., Itodo E., Faleye O. (2025). Machine learning for anomaly detection in smart grid energy consumption: A one-class SVM approach. FUTA J. Eng. Eng. Technol..

[91] Elgarhy I., Badr M.M., Mahmoud M., Alsabaan M., Alshawi T., Alsaqhan M. (2024). Xai-based accurate anomaly detector that is robust against black-box evasion attacks for the smart grid. Appl. Sci..

[92] Din G.M.U., Mauthe A.U., Marnerides A.K. Appliance-level short-term load forecasting using deep neural networks. Proceedings of the 2018 International Conference on Computing, Networking and Communications (ICNC).

[93] Jiang H., Yan Q., Jiang Z. (2024). Symmetric positive definite autoencoder method for multivariate time series anomaly detection. J. Comput. Appl..

[94] Huang J., Qiu Y. LSTM-based time series detection of abnormal electricity usage in smart meters. Proceedings of the 2025 5th International Symposium on Computer Technology and Information Science (ISCTIS).

[95] Bai S., Kolter J.Z., Koltun V. (2018). An empirical evaluation of generic convolutional and recurrent networks for sequence modeling. arXiv.

[96] Lee S., Jin H., Nengroo S.H., Doh Y., Lee C., Heo T., Har D. Smart metering system capable of anomaly detection by bi-directional LSTM autoencoder. Proceedings of the 2022 IEEE International Conference on Consumer Electronics (ICCE), Virtual, 7–9 January 2022.

[97] Rahman H., Sheikh N.U., Nanda P. (2025). Hybrid CNN-LSTM based anomaly detection for energy theft in residential smart meter data. 2025 9th Cyber Security in Networking Conference (CSNet).

[98] Pestana R., Costa P., Nie Q., Geraldes C., Leandro C., Silva R., Hu H., Cardenas M. (2025). Technology-divergent anomaly detection in smart meters: Adaptive LSTM-autoencoders for digital vs. analog error signals. 2025 5th International Conference on Electrical, Computer, Communications and Mechatronics Engineering (ICECCME).

[99] Beily M.D.E., Darwish I., Mohamed A., Sawon K., Shafikuzzaman M. (2024). A deep-learning approach for detecting anomalies in commercial building load profiles. 2024 IEEE 19th Conference on Industrial Electronics and Applications (ICIEA).

[100] Schlegl T., Seeböck P., Waldstein S.M., Langs G., Schmidt-Erfurth U. (2019). F-AnoGAN: Fast unsupervised anomaly detection with generative adversarial networks. Med. Image Anal..

[101] Li X., Zhang W., Ding Q. (2019). Cross-domain fault diagnosis of rolling element bearings using deep generative neural networks. IEEE Trans. Ind. Electron..

[102] Liu J., Li C., Su Y., Sun X. (2022). A study on bearing fault diagnosis based on LSGAN-SqueezeNet. J. Vib. Shock.

[103] Mallola A., Prata A.R., Preethi M.S. (2024). A novel approach for developing a framework for enhanced energy consumption management using CGAN. 2024 International Conference on Cybernation and Computation (CYBERCOM).

[104] Mustafa G., Nawaz A., Abbas M.A., Babar M., Khan Z. (2026). TRANS-DEEP: An optimized deep learning framework for electricity theft detection in smart grids. PeerJ Comput. Sci..

[105] Nandhini N., Manikandan V., Elango S. (2025). An interpretable generalized additive neural networks for electricity theft detection in smart cities using balanced data and intelligent grid management. Energy Build..

[106] Lou L., Liu Z. (2025). A multi-dimensional telemetry data pattern mining method based on matrix profile. Telecommun. Eng..

[107] Stamatescu G., Plamanescu R., Ciornei I., Albu M. Detection of anomalies in power profiles using data analytics. Proceedings of the 2022 IEEE 12th International Workshop on Applied Measurements for Power Systems (AMPS).

[108] Hussain S., Mustafa M.W., James S.E., Baloch S.K. (2022). Electric theft detection using unsupervised machine learning-based matrix profile and K-means clustering technique. Computational Intelligence in Machine Learning: Proceedings of the 2nd International Conference on Computational Intelligence in Machine Learning (ICCIML 2022).

[109] Wang J., Chen Y., Geng R., Zhao W., Pan Z., Fu C., Wang X., Chen T., Cai Y., Du H. (2024). An efficient anomaly detection model of power consumption behavior for industrial customers. 2024 11th International Conference on Behavioural and Social Computing (BESC).

[110] Stamatescu G., Plamanescu R., Albu M. (2025). Online embedded analytics for energy time series pre-processing in LVDC microgrids. 2025 IEEE Seventh International Conference on DC Microgrids (ICDCM).

[111] Zhang C., Sun J., Lu R., Wang P. Anomaly detection model of power grid data based on STL decomposition. Proceedings of the 2021 IEEE 5th Information Technology, Networking, Electronic and Automation Control Conference (ITNEC).

[112] Liu J., Wu S., Cao W., Guo Y., Gong S. (2021). Smart grid data anomaly detection method based on cloud computing platform. 7th International Conference on Artificial Intelligence and Security (ICAIS 2021).

[113] Shiwei C., Lei S., Ying S. (2025). A hybrid approach for anomaly detection and correction of power data in modern power systems. 2025 8th International Conference on Power and Energy Applications (ICPEA).

[114] Kwon H.-R., Kim P.-K. (2021). A missing data compensation method using LSTM estimates and weights in AMI system. Information.

[115] Schaffer M., Tvedebrink T., Marszal-Pomianowska A. (2022). Three years of hourly data from 3021 smart heat meters installed in Danish residential buildings. Sci. Data.

[116] Sousa J.C., Bernardo H. (2022). Benchmarking of load forecasting methods using residential smart meter data. Appl. Sci..

[117] Zhang H., Wang H., Ding Y. (2024). Missing power data restoration method based on DA multiple imputation and power internet of things. Electron. Des. Eng..

[118] Dahale S., Natarajan B. (2021). Multi time-scale imputation aided state estimation in distribution system. 2021 IEEE Power & Energy Society General Meeting (PESGM).

[119] Madbhavi R., Natarajan B., Srinivasan B. (2022). Meter placement approaches for matrix completion-based distribution system state estimator. Electr. Power Syst. Res..

[120] Liu R., Pan F., Yang Y., Hong W., Li Q., Lin K., Liu S. (2023). Theoretical line loss calculation method for low-voltage distribution network via matrix completion and ReliefF-CNN. Energy Rep..

[121] Ma Y., Liu J., Chen C., Xu G., Ma X. (2025). Matrix filling-based power quality data restoration system for power internet of things. J. Comb. Math. Comb. Comput..

[122] Cai L., Gu J., Jin Z. Low rank matrix completion for recovering missing load data in power system. Proceedings of the 2018 Chinese Automation Congress (CAC).

[123] Donti P.L., Liu Y., Schmitt A.J., Bernstein A., Yang R., Zhang Y. (2020). Matrix completion for low-observability voltage estimation. IEEE Trans. Smart Grid.

[124] Luo Y., Cai X., Zhang Y., Xu J., Yuan X. Multivariate time series imputation with generative adversarial networks. Proceedings of the Advances in Neural Information Processing Systems (NeurIPS 2018).

[125] Qian Y., Tian L., Zhai B., Zhang S., Wu R. (2022). Informer-WGAN: High missing rate time series imputation based on adversarial training and a self-attention mechanism. Algorithms.

[126] Kong H., Li X., Li H., Xiao Y., Ye S., Zhang C. (2026). GAN-Transformer for multivariate power consumption imputation. KSII Trans. Internet Inf. Syst..

[127] Jalalifar R., Delavar M.R., Ghaderi S.F., Alehashem S.L.M. (2025). SAGANConvLSTM: A novel spatio-temporal forecasting approach combining semivariogram-enhanced GAN and ConvLSTM for power load forecasting. Comput. Electr. Eng..

[128] Hwang J., Suh D. (2024). CC-GAIN: Clustering and classification-based generative adversarial imputation network for missing electricity consumption data imputation. Expert Syst. Appl..

[129] Zhang S., Deng S., Liu Q. (2026). The missing data recovery method based on improved GAN. Comput. Mater. Contin..

[130] de Rezende J.A., Teixeira E.H., Pimenta T.C. (2026). Design of experiments for Pix2Pix hyperparameter analysis in energy consumption data imputation. 2026 International Conference on Artificial Intelligence, Computer, Data Sciences and Applications (ACDSA).

[131] Sun H., Ma F., Yan C. (2026). Prediction of missing power quality data in V2G based on ARIMA model. J. Qufu Norm. Univ. (Nat. Sci. Ed.).

[132] An Y., Zhu Y. (2021). Prediction of power load based on linear regression and exponential smoothing methods. Power Equip. Manag..

[133] Vannisa V.S., Hartono J., Supriyadi G., Munir B.S. (2025). Comparative study of prediction methods for estimating missing data in advanced metering infrastructure system in Indonesia. 2025 International Conference on Advanced Technologies in Energy and Informatic (ICATEI).

[134] Supriyadi G., Hartono J., Alam A.S. (2025). LSTM methods for estimating missing data in advanced metering infrastructure (AMI) in Indonesia. 2025 IEEE International Conference on Industry 4.0, Artificial Intelligence, and Communications Technology (IAICT).

[135] Raghuvamsi Y., Teeparthi K. (2024). Distribution system state estimation with Transformer-Bi-LSTM-based imputation model for missing measurements. Neural Comput. Appl..

[136] Wu Y., Zhang Y., Peng H., Liu Z., Yang Y., Dai S., Li Q. (2025). Analysis and processing of on-site operation data of smart meters and basic error prediction. 2025 2nd International Symposium on IoT and Intelligent Robotics (IoTIR).

[137] Utomo D., Hsiung P.-A. Anomaly detection at the IoT edge using deep learning. Proceedings of the 2019 IEEE International Conference on Consumer Electronics—Taiwan (ICCE-TW).

[138] Jabbari Zideh P.C.M., Srivastava A.K. (2024). Physics-informed machine learning for data anomaly detection, classification, localization, and mitigation: A review, challenges, and path forward. IEEE Access.

[139] Brown C.M.J.R., Reeves A.H. (2022). Longitudinal changes in residential electricity consumption patterns during and after COVID-19 lockdowns. Nat. Energy.

[140] Clark J., Liu Z., Japkowicz N. Adaptive threshold for outlier detection on data streams. Proceedings of the 2018 IEEE 5th International Conference on Data Science and Advanced Analytics (DSAA).

[141] Cappello L., Padilla O.H.M. (2025). Bayesian variance change point detection with credible sets. IEEE Trans. Pattern Anal. Mach. Intell..

[142] Berrisford A.J. Revised IEEE-1459 power definitions: A revenue metering viewpoint. Proceedings of the 2024 IEEE International Instrumentation and Measurement Technology Conference (I2MTC).

[143] Malengret M., Gaunt C.T., Adams S., Oyedokun D.T.O. Accurate metering of apparent power based on general power theory. Proceedings of the 2018 IEEE PES/IAS PowerAfrica.

[144] Fahim S.R., Avro S.S., Sarker S.K., Rokunuzzaman M., Das S.K. Improved differential strobe terminal based open-loop control of smart power factor metering system. Proceedings of the 2019 1st International Conference on Advances in Science, Engineering and Robotics Technology (ICASERT).

[145] Dong Y. Correlation-driven multi-level multimodal learning for anomaly detection on smart electric grid. Proceedings of the 2025 2nd International Conference on Smart Grid and Artificial Intelligence (SGAI).

[146] Song Q., Yang J., Fu Q., Zhang S., Shi K., Zhang H. A Transformer-based multi-modal fault diagnosis model for power metering terminals. Proceedings of the 2025 5th International Conference on New Energy and Power Engineering (ICNEPE).

[147] Zhu Y., Cui Y., Wu Y., Li D. Metering asset fault perception and state assessment model fusing multi-modal visual learning. Proceedings of the 2025 7th International Conference on Electronics and Communication, Network and Computer Technology (ECNCT).

[148] Acevedo E., Schmidt M., Silva S., Mariño C., Gomez Á., Fernández A., Martins A., Caudullo G., Berois M. NTL detection: Multi-modal architecture for smart meter fraud detection in a realistic deployment. Proceedings of the 2025 IEEE PES Innovative Smart Grid Technologies Conference—Latin America (ISGT LA).

[149] Olojede D.R., Uddin M.J., Jacob R.A., Coskunuzer B., Zhang J. (2026). Topology informed transformer for cyber attack detection in grid-connected PV systems. IEEE Trans. Sustain. Energy.

[150] Yuan Y., Dehghanpour K., Wang Z., Bu F. (2022). Multisource data fusion outage location in distribution systems via probabilistic graphical models. IEEE Trans. Smart Grid.

[151] Jing S., Wu J., Zhan P., Zhang T. RFETR: Residual frequency-enhanced two-stage reconstruction for time series anomaly detection. Proceedings of the 2026 International Conference on Communication Networks and Machine Learning (CNML).

[152] Zheng Z., Liu W., Liu R., Wang L., Mao L., Qiu Q., Ling G. (2022). Anomaly detection of metro station tracks based on sequential updatable anomaly detection framework. IEEE Trans. Circuits Syst. Video Technol..

[153] Wang Y., Zhong X., Shao Y., Hu J., Xu T., Bao Y., Li W. Multi-scale time series ensemble learning for information system anomaly detection. Proceedings of the 2023 IEEE Smart World Congress (SWC).

[154] Li H., Wu Z., Chen W., Yang X. A multi-scale adaptive sliding window-based algorithm for anomaly detection in meter-generated metering data streams. Proceedings of the 2024 Boao New Power System International Forum—Power System and New Energy Technology Innovation Forum (NPSIF).

[155] Yao Y., Ding F., Liu W. A hybrid data-driven and model-based anomaly detection scheme for DER operation. Proceedings of the 2022 IEEE Power & Energy Society Innovative Smart Grid Technologies Conference (ISGT).

[156] Wang B., Li Z., Li W., Wang Z. (2026). Integrated distribution-free detection and adaptive robust scheduling for V2G smart home microgrids. IEEE Trans. Consum. Electron..

[157] Klusacek J., Drapela J., Langella R., Meyer J. Revenue metrics ability to correctly register bidirectional active energy flows in active distribution networks. Proceedings of the 2023 IEEE 13th International Workshop on Applied Measurements for Power Systems (AMPS).

[158] Zhang Y., Zhang T., Li G., Zhou M., Zhou Q. (2026). Adaptive scheduling of distribution networks with massive DERs and EVs: A constraint-guided deep reinforcement learning approach. Int. J. Electr. Power Energy Syst..

[159] Han F., Wang X., Chen Y., Qiao J., Zhu Q., Feng M. A data and model driven method for low-voltage distribution area topology identification. Proceedings of the 2024 9th Asia Conference on Power and Electrical Engineering (ACPEE).

[160] Xu J., Sha F., Sun C., Li R. Phase and hierarchical topology identification in low-voltage distribution areas. Proceedings of the 2025 IEEE PES Innovative Smart Grid Technologies—Asia (ISGT Asia).

